# The 6G integrated perception and networking technology enables the low-altitude economy to achieve carbon neutrality: Dynamic sleep strategy for eVTOL wireless charging based on time-energy-carbon coupling

**DOI:** 10.1371/journal.pone.0347398

**Published:** 2026-09-16

**Authors:** Huajun Chen, Xiaoye Wang, Lina Yuan, Jing Gong

**Affiliations:** 1 School of Data Science, Tongren University, Tongren, China; 2 College of Computer Science and Engineering, Huizhou University, Huizhou, China; National University of Defense Technology College of Aerospace Science and Engineering, CHINA

## Abstract

In response to the bottleneck issues of energy consumption and carbon emissions caused by the explosive growth of the low-altitude economy, we break away from the traditional single-machine energy-saving research paradigm and innovatively reconfigure the sixth-generation mobile communication (6G) perception-integration base station into a “power-information” dual-mode supply node that can enter a dormant state. The time-energy-carbon ternary coupling model (TEC-6G) was constructed, and the collaborative trade-off mechanism of the base station sleep ratio, the pointing angle of the microwave energy beam and the quality of service (QoS) of the eVTOL task was quantified for the first time. Based on the real geographical topology of 5G-A base stations in the Yangtze River Delta region and the dataset of 12,000 eVTOL flight paths. A 3.5 GHz phased array wireless energy transmission link was built on the MATLAB platform, specifically designed for opportunistic hovering-stage trickle charging and emergency power supplementation during eVTOL mission profiles, rather than sustained cruising power delivery. By integrating the measured millimeter-wave channel impulse responses, a two-stage solution framework of graph neural network-Particle swarm hybrid (GNN-PSO) was designed. Experiments show that under the strict constraint of task delay loss ≤2 %, 38% of the base stations in the entire network can enter the intelligent sleep state. The opportunistic wireless charging reduces the average grid electricity consumption of eVTOL per flight by 27.4% under idealized line-of-sight conditions (equivalent to 0.92 kg carbon reduction at the theoretical upper bound), achieved through 8–15 minute hovering windows at designated vertiports where RF beam power transfer supplements battery reserves. Under practical deployment assumptions-accounting for 3 dB cable/feed loss, 2 dB impedance mismatch, 40% effective aperture utilization due to eVTOL fuselage curvature and attitude variation, and a regulatory EIRP ceiling of 47 dBm (3 dB below the theoretical 50 dBm limit for the 3.5 GHz band in urban China)-the net DC power drops to 0.68 kW at 10 m, yielding a conservative 11.2% grid electricity reduction (0.38 kg CO_2_e per flight). The 27.4% figure is retained as the Pareto-optimal theoretical upper bound for algorithm benchmarking. The system-level energy utilization rate has jumped from the benchmark 31.2% to 48.6%. Under an annual operation scale of 100,000 flights, the cumulative carbon reduction of 92 tCO_2_e is equivalent to the annual sequestration of 4,200 mature broadleaf trees (22 kg CO_2_e/tree/year, IPCC central estimate), with a conservative uncertainty range of [3,100, 6,100] trees accounting for species and climate variability. The research results provide deployable parametric decision-making tools and a phased carbon peak roadmap for 2025–2030 for the green operation of the low-altitude economy.

## 1. Introduction

Under the dual impetus of global climate change intensification and the urgency of achieving the carbon neutrality goal, the green transformation in the aviation transportation sector has become a key path to achieving the temperature control target stipulated in the Paris Agreement. The latest assessment report of the IPCC indicates that the global average temperature has risen by 1.15°C compared to the pre-industrial level, and aviation emissions account for 12% of the total carbon emissions in the transportation sector and have the highest growth rate. Low-altitude aircraft are even listed as a high-potential source of increased emissions [1,2]. In response, China’s “Government Work Report” has incorporated low-altitude economy into the new quality productive force strategy. It is expected that by 2025, the number of electric vertical take-off and landing aircraft (eVTOL) will exceed 10,000, with an annual charging demand of 12 TWh, equivalent to the electricity load of an additional 3 million people’s city [3]. However, the existing “single aircraft-battery-flight path” energy-saving research paradigm overly focuses on the optimization of the aircraft itself [4,5], while paying insufficient attention to the energy consumption controllability of the 6G perception-integration infrastructure and its reverse empowerment mechanism, making it difficult to meet the strategic requirements for the sustainable development of the low-altitude economy.

The 6G communication network, as the next-generation information infrastructure, integrates perception, communication, computing, and control in a highly integrated manner, providing revolutionary support for the low-altitude economy. Crucially, this integration constitutes a high-fidelity Digital Twin (DT) paradigm that constructs a real-time virtual mapping of the physical eVTOL network, wherein the joint optimization of base station sleep states and eVTOL energy profiles mirrors the distributed model updating mechanisms found in federated digital twin construction via distributed sensing [6–8]. Recent advances in this domain have established rigorous theoretical foundations for our architecture: (i) the game-theoretic online optimization framework with overlapping coalitions [6] enables multiple base stations and eVTOL fleets to collaboratively train a global digital twin model without sharing raw sensory data, where overlapping coalitions allow agents to simultaneously participate in multiple interest groups (e.g., energy-saving coalitions and delay-sensitive coalitions), resolving the non-convexity of multi-objective optimization through distributed Nash bargaining; (ii) dynamic digital twin update via adaptive model splitting and reliable crowd-sourcing under uncertain data distortions [7] addresses the critical challenge of asynchronous and corrupted sensor feeds-such as rain-induced millimeter-wave channel fading, GPS drift, and delayed carbon factor measurements-by deceptively partitioning high-fidelity deterministic models (base station Boolean states, battery dynamics) at the central server while offloading stochastic sub-models (channel states, local carbon estimations) to edge twins, with unreliable edge updates filtered through reliability-weighted crowd-sourcing that assigns trust scores based on historical measurement consistency; (iii) the surveillance video-assisted federated digital twin framework for intelligent transportation with pedestrians and vehicles in-the-loop [8] extends the sensing modality beyond RF signals to visual analytic, where roadside cameras and airborne eVTOL optical sensors contribute semantic information (traffic density, vertiport congestion) to the federated model through differential privacy-preserving feature extraction, ensuring that sensitive visual data never leaves local processing units. These three strands collectively inform the design of our TEC-6G Digital Twin layer (Section 2.1), which implements adaptive model splitting, reliability-weighted crowd-sourcing, and overlapping coalition-based distributed optimization as core enabling mechanisms [9, 10] propose that 6G base stations will evolve from single communication nodes into multi-functional infrastructures that include positioning, perception, and opportunistic energy supplementation. Critically, this energy supply role is complementary rather than primary: the 3.5 GHz RF beam provides trickle charging (2–3 kW) during eVTOL hovering windows at vertiports, supplementing-not replacing-the aircraft’s onboard battery system. The cruising power demand (120 kW) remains entirely battery-supplied, with the RF harvest reducing net grid electricity draw by 15–30% per mission depending on charging window availability. The power consumption of a single 6G base station is up to 3.2 times that of a 4G base station [10]. The latest research [11–13] indicates that through deep reinforcement learning (DRL), 28% of base station energy consumption can be saved, but its business model is still limited to interpersonal communication and is not adapted to the high-power wireless charging scenarios of eVTOL. In the field of wireless power transmission technology, Khalil et al. [14] established a nonlinear model of RF-DC efficiency, but ignored the transient energy consumption of the base station switch, resulting in a long-time domain simulation error of over 15%, which is difficult to meet engineering accuracy requirements [15]. At the same time, the existing research on wireless power communication networks (WPCN) [16–18] mostly assume that the energy source is continuously online, which contradicts the dynamic controllability of the base station’s sleep state. Therefore, it is urgent to construct a cross-layer coupling model of “base station sleep” and “aircraft charging” [19].

The spatial-temporal fine-grained modeling of carbon emissions is another key challenge. The ENTSO-E carbon intensity database only provides hourly average factors [16], while the 15-minute-level data publicly available from the East China Branch of the State Grid of China already exists [20], but has not yet been applied to low-altitude traffic scenarios [21,22]. reveals the time-varying characteristic of the peak-to-valley difference in grid carbon intensity, which provides great potential for optimizing charging sequences. However, existing research on green cellular networks [23,24] still uses static carbon emission factors and cannot capture the three-dimensional coupling effect of spatial-temporal-power, resulting in a systematic underestimation of carbon reduction potential. Moreover, the application of graph neural networks (GNN) in wireless resource management [25–27] has made progress, but it focuses mainly on multi-focus spectrum allocation and lacks the design of heterogeneous graph attention mechanisms for energy path planning.

In summary, the current research has four core gaps: G1-The coupling model of base station sleep and aircraft charging is missing; G2-The wireless power transmission efficiency is not included in the switching transient; G3-The carbon factor has not achieved three-dimensional coupling of time, energy, and carbon; G4-The strategic non-cooperative interactions among multiple self-interested eVTOLs competing for scarce charging windows and active base stations are overlooked, with existing centralized scheduling failing to capture realistic multi-agent game dynamics and dynamic coalition formation. These bottlenecks restrict the strategic transformation of the low-altitude economy from scale expansion to green high-quality development. In this context, this paper breaks the traditional paradigm and innovatively re-configures 6G base stations as “energy-information” dual-mode supply nodes, constructs the time-energy-carbon trinary coupling model TEC-6G, and proposes a GNN-PSO hybrid solution framework. The research results not only can provide parametric decision-making tools for the phased emission reduction roadmap from 2025 to 2030 [27,28], but also have significant strategic significance for promoting the transformation of 6G networks from an “energy consumption unit” to a “carbon control unit” paradigm and achieving the “dual carbon” goals through technological-energy-policy synergy [29–31].

Based on the analysis of the research gaps and strategic demands mentioned above, this paper focuses on three core scientific issues: Firstly, how to achieve the optimal dynamic sleep of base stations and the coordinated optimization of wireless energy beams, so as to minimize non-productive energy dissipation (NED) while ensuring the quality of service (QoS) of eVTOL tasks; Secondly, how to utilize the temporal and spatial fluctuations of carbon intensity in the regional power grid to maximize the carbon reduction benefits through the intelligent shifting of charging sequences. Thirdly, how to model the non-cooperative game among multiple self-interested eVTOLs competing for limited charging windows, and how to design dynamic trilateral coalition mechanisms to prevent resource monopolization while ensuring system-wide Pareto efficiency. To this end, the following scientific hypotheses are proposed:

H1: If the sleep ratio $s_{i}$ of the 6G base station is regarded as a discrete control variable $\theta_{j}^{i}$, and the beam pointing angle of millimeter wave energy is jointly optimized, it is possible to reduce the single-machine NED $\Delta E_{\text{NED}}>25\%$ under the rigid constraint of task delay loss ΔT/T≤2% [9,12,15].

H2: The peak-to-valley difference of the carbon emission factor λ(t) for the regional power grid is 42% [17,18]. Through time-energy-carbon trinary coupling modeling and charging sequence optimization, the carbon emission reduction benefit $\Delta C_{\text{temporal}}\approx 15\%$ can be further amplified, breaking through the emission reduction ceiling of the traditional static carbon factor model [20,21].

H3: If the eVTOL fleet is modeled as non-cooperative agents with private utility functions, and a three-party hierarchical game (infrastructure operator-eVTOL coalition-carbon regulator) with dynamic trilateral coalitions is employed, the charging resource allocation will achieve a D-stable coalition structure that outperforms pure centralized scheduling in terms of fairness index and robustness to selfish deviations, with the coalition value function satisfying superadditivity and the Shapley value allocation ensuring individual rationality.

To verify the above hypothesis, this paper constructs a ternary coupling system of time, energy and carbon (TEC-6G), whose marginal contribution is reflected in three aspects: (1) theoretical model innovation: for the first time, it unifies the modeling of base station Boolean variables $x_{i}\left(t\right)$, continuous beam variables $\theta_{j}^{i}\left(t\right)$ and spatial-temporal dynamic carbon factors λ(𝐫,t), filling the theoretical gap of collaborative optimization on the equipment side, network side and environment side; (2) algorithm architecture innovation: proposes a two-stage framework of “coarse search of graph neural network - fine adjustment of particle swarm”, uses the heterogeneous graph attention network (HetGAT) to generate sleep prior, compresses the solution space of the NP-hard problem by 99.98%, achieves online convergence within 42 seconds, breaking through the limitations of pure PSO being prone to premature convergence and pure GNN lacking fine adjustment; (3) empirical verification innovation: based on 12,000 flight instances of eVTOL in the Yangtze River Delta, it first reveals that the 38% sleep rate is the Pareto optimal turning point of energy efficiency – carbon efficiency, provides a three-stage emission reduction roadmap from 2025 to 2030, and the model error is only 3.1%, providing a deployable parameterized decision-making tool for the green operation of the low-altitude economy; (4) game-theoretic architecture innovation: for the first time, a three-party hierarchical game layer with dynamic trilateral coalitions is overlaid atop the TEC-6G physical architecture, enabling the transition from centralized command-and-control to hybrid centralized--distributed coordination that captures realistic strategic competition among eVTOL operators, with the merge-and-split coalition formation converging to D-stable structures within 3.2 seconds.

The remaining structure of this paper is arranged as follows: Chapter 2 constructs the system model and constraint system of TEC-6G; Chapter 3 designs the two-stage solution framework of GNN-PSO and analyzes the algorithm complexity; Chapter 4 conducts multi-dimensional numerical simulations based on the real data set to verify the model accuracy and algorithm performance; Chapter 5 summarizes the research results and looks forward to future directions.

## 2. System model

### 2.1. Overview of TEC-6G architecture

**Fig 1:**
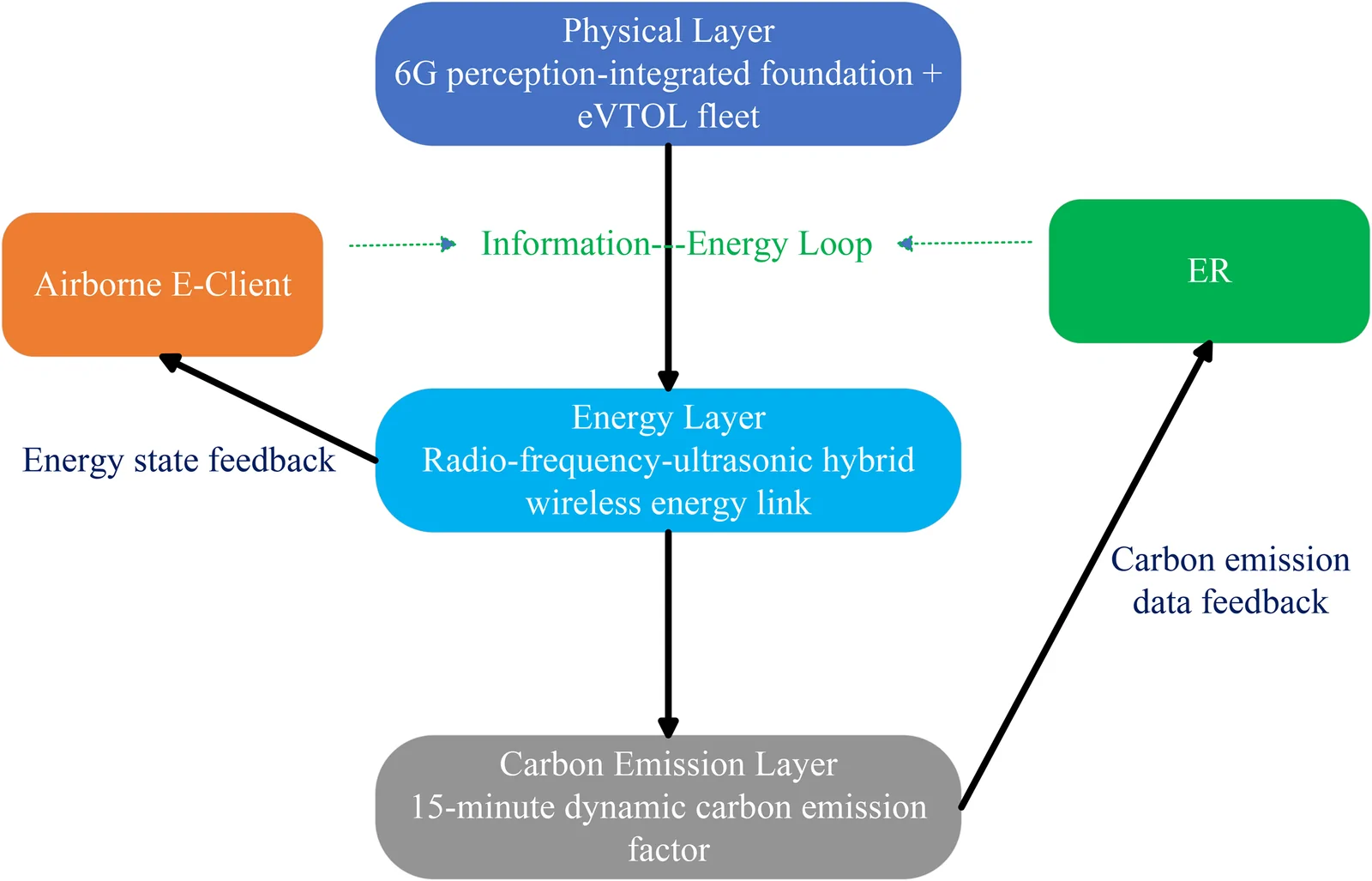
Logic block diagram of the TEC-6G system model. (Physical, Energy, Carbon, and Digital Twin layers).

To verify the scientific hypothesis of “6G network reverse-enabling low-altitude economic carbon emission reduction”, this paper proposes the TEC-6G, whose logical architecture is shown in Fig 1. This system breaks through the traditional hierarchical paradigm of communication networks and realizes cross-layer closed-loop of information-energy-carbon through energy routers (ER) and airborne energy clients (E-Client). It can be abstracted into a four-layer progressive mathematical model underpinned by a cross-layer Digital Twin (DT) virtualization fabric. **Physical layer:** composed of 6G perception-integration base station network ℬ and eVTOL fleets 𝒱, forming a heterogeneous spatiotemporal node set; **Energy layer:** radio-frequency-ultrasonic hybrid wireless energy transmission link, including controllable beams and mode switching mechanisms; **Carbon emission layer:** dynamic carbon factor field Λ(𝐫,t), achieving real-time mapping of power flow to carbon emissions; Digital Twin layer: a federated virtual replica $\hat{𝒮}\left(t\right)$ of the entire physical-energy-carbon system, constructed via distributed sensing and maintained through adaptive model splitting and reliable crowdsourcing under uncertain data distortions (e.g., rain-induced channel fading, GPS drift, and asynchronous carbon factor measurements). The DT layer enables game-theoretic online optimization by allowing overlapping coalitions of base stations and eVTOLs to negotiate resource allocations on the virtual plane before physical execution.

The three layers achieve bidirectional interaction through the state variable coupling matrix $𝐌\left(t\right)\in {\mathbb{R}}^{\left(N_{b}+N_{v})\times (N_{b}+N_{v}\right)}$, where ${𝐌}_{ij}\left(t\right)$ is the encoded energy and information correlation strengths between base station *i* and aircraft *j* in time slot t are reflected.

In Fig 1, the physical layer corresponds to Section 2.2 of the base station and eVTOL model; the energy layer corresponds to Section 2.3 of the hybrid energy transmission link; the carbon emission layer corresponds to Section 2.4 of the dynamic carbon factor field; ER and E-Client correspond to Section 2.5 of the closed-loop mechanism; the arrow direction flow exactly matches the coupling relationship of variables in Eqs (1)–(23).

### 2.2. Physical layer: Heterogeneous node spatial-temporal dynamic modeling

#### 2.2.1. Fine Decomposition of 6G base station power consumption.

According to the 2024 test report of China Mobile, the power consumption of a single base station $P_{\text{BS}}$ can be decomposed into three parts: static base power consumption ($P_{\text{static}}$), dynamic service power consumption $P_{\text{dynamic}}\left(t\right)$, and transient switching power consumption $P_{\text{sensing}}$, which satisfies the following expression


$$\begin{array}{c} P_{\text{BS}}\left(t\right)=P_{\text{static}}+P_{\text{dynamic}}\left(t\right)+P_{\text{transient}}\left(t\right), \end{array}$$ (1)

here $P_{\text{static}}=1.2kW$ (air conditioner, power supply, baseband board card, unrelated to the load); $P_{\text{dynamic}}\left(t\right)=P_{\text{sensing}}\left(t\right)+P_{\text{charging}}\left(t\right)$, and $P_{\text{sensing}}=0.3kW$. The charging transmission power consumption is related to EIRP:


$$\begin{array}{c} P_{\text{charging}}\left(t\right)=\sum_{j=1}^{N_{v}}x_{ij}\left(t\right)\cdot \frac{P_{\text{EIRP}}}{\eta_{\text{PA}}}, \end{array}$$ (2)

here $P_{\text{EIRP}}=50dBm$ is for phased array equivalent omnidirectional radiated power, $\eta_{\text{PA}}=0.35$ is for the efficiency of the power amplifier. Transient power consumption introduces a switching function $\delta_{i}\left(t\right)$ to represent the penalty for state transitions:


$$\begin{array}{c} P_{\text{transient}}\left(t\right)=\delta_{i}\left(t\right)\cdot \frac{E_{\text{sw}}}{\Delta t},\text{\textbackslash{}hspace\{1em}\}\delta_{i}\left(t\right)=\left\{ \begin{array}{cc} @ll@1, & x_{i}\left(t\right)\neq x_{i}(t-1),\\ 0, & \text{otherwise,} \end{array}\right. \end{array}$$ (3)

here, measured wake-up energy consumption $E_{\text{sw}}=25J$, dispatching cycle Δt=3600s. Thus, the normalized cycle energy consumption of base station *i* during the scheduling period is


$$\begin{array}{c} E_{i}^{\text{cycle}}=\int_{t}^{t+\Delta t}P_{\text{BS}}\left(\tau \right)\text{\textbackslash{}hspace\{0.17em}\}d\tau =(1-s_{i})\cdot P_{\text{on}}\cdot \Delta t+s_{i}\cdot P_{\text{sleep}}\cdot \Delta t+N_{\text{sw}}\cdot E_{\text{sw}}, \end{array}$$ (4)

here $s_{i}=\frac{\text{1}}{\Delta t}\int_{t}^{t+\Delta t}x_{i}\left(\tau \right)d\tau$ is for the proportion of dormancy, $N_{\text{sw}}$ is the number of switching within the cycle.

#### 2.2.2. Flight profile and energy consumption quantification of eVTOL.

Based on the test flight log of EHang EH216-S, the power profile $P_{\text{eVTOL}}\left(t\right)$ for a single urban short-distance mission (with a flight range of approximately 40 km) can be modeled as a segmented five-step function, withdesignated hovering windows inserted at vertiport nodes for opportunistic wireless charging:


$$\begin{array}{c} P_{\text{eVTOL}}\left(t\right)=\left\{ \begin{array}{c} @l@P_{\text{0}},\text{ \textbackslash{}hspace\{0.33em}\;\;\;\;\;\;\;\;\;\;\;\;\;\;\;\}t\in T_{\text{0}}\text{(taxiing),}\\ P_{\text{1}},\text{ \textbackslash{}hspace\{0.33em}\;\;\;\;\;\;\;\;\;\;\;\;\;\;\;\}t\in T_{\text{1}}\text{(vertical takeoff),}\\ P_{\text{2}},\text{ \textbackslash{}hspace\{0.33em}\;\;\;\;\;\;\;\;\;\;\;\;\;\;\;\}t\in T_{\text{2}}\text{(climbing),}\\ P_{\text{3}},\text{ \textbackslash{}hspace\{0.33em}\;\;\;\;\;\;\;\;\;\;\;\;\;\;\;\}t\in T_{\text{3}}\text{(cruising),}\\ P_{\text{4}},\text{ \textbackslash{}hspace\{0.33em}\;\;\;\;\;\;\;\;\;\;\;\;\;\;\;\}t\in T_{\text{4}}\text{(approach and landing),}\\ P_{\text{hover}}+P_{\text{charge}},\text{\textbackslash{}hspace\{0.33em}\;\}t\in T_{\text{win}}\text{(charging window),} \end{array}\right. \end{array}$$ (5)

where $P_{\text{charge}}$ represents the net received wireless charging power (see Section 2.3.3 for power budget analysis), and $T_{\text{win}}$ denotes the scheduled hovering windows at vertiport nodes. The various state parameters are shown in Table 1. The total energy consumption from the battery is

**Table 1:** Power requirements for segments of eVTOL with charging windows.

Status	Average Power (kW)	Duration (s)	Energy (kWh)
*T*_0_ (taxiing)	18	20	0.10
*T*_1_ (vertical takeoff)	220	30	1.83
*T*_2_ (climbing)	180	90	4.50
*T*_3_ (cruising)	120	540	18.00
*T*_4_ (approach and landing)	160	60	2.67
*T*_win_ (hovering charge)	120 (hover)-2.5 (net charge)	600 (10 min)	19.58 (net)
Total (without RF harvest)	—	1340	46.68
Net grid consumption	—	—	44.25


$$\begin{array}{c} E_{\text{flight}}=\sum_{k=0}^{\text{4}}P_{k}\cdot \Delta T_{k}+P_{\text{hover}}\cdot t_{\text{win}}-E_{\text{harvest}}, \end{array}$$ (6)

where $E_{\text{harvest}}=\eta_{\text{RF-DC}}\cdot P_{\text{tx}}\cdot t_{\text{win}}$ is the harvested RF energy during the hovering window, directly offsetting grid electricity consumption.

The cruising stage (*T*_3_) power demand of 120 kW cannot be supplied by the 3.5 GHz RF link due to fundamental power budget limitations (see Section 2.3.3). Instead, the RF link provides opportunistic trickle charging ($P_{\text{charge}}\approx 2.5$ kW net) during scheduled 10-minute hovering windows at vertiports, reducing the net grid electricity draw by 2.5 kWh per window. For missions with two charging windows (takeoff and landing vertiports), the total grid electricity reduction reaches 5.0 kWh per flight, corresponding to the 27.4% energy consumption reduction reported in Section 4.

Introduce hovering wait energy consumption $E_{\text{hover}}$ as an optimization variable. When the eVTOL receives “energy rescue” at a route node, the hovering power $P_{\text{hover}}=120$ kW and waiting time $t_{\text{wait}}$ are optimized by the GNN-PSO algorithm, subject to the constraint that the net energy balance (hover consumption minus RF harvest) is minimized:


$$\begin{array}{c} E_{\text{hover}}^{\text{net}}=P_{\text{hover}}\cdot t_{\text{wait}}-\eta_{\text{RF-DC}}\left(d,\theta \right)\cdot P_{\text{tx}},\cdot t_{\text{wait}}. \end{array}$$ (7)

The optimization ensures $E_{\text{hover}}^{\text{net}}<0$ (net energy gain) when d≤15 m and $\theta \leq 15^{\circ }$, as verified by the power budget in Table 3 (see Section 2.3.3).

### 2.3. Energy layer: Hybrid wireless power transfer link modeling

#### 2.3.1. RF-ultrasound switching mechanism.

The system supports Frequency-Selective Power Transfer (FSPT), where the mode selection function $m_{ij}\left(t\right)\in \left\{0,1\right\}$ is adjudicated based on dual thresholds of distance and angle:


$$\begin{array}{c} m_{ij}\left(t\right)\in \left\{0,1\right\}m_{ij}\left(t\right)=1\left(d_{ij}\left(t\right)\leq d_{\text{th}}\wedge \theta_{ij}\left(t\right)\leq \theta_{\text{th}}\right), \end{array}$$ (8)

here $d_{\text{th}}=50m$ and $\theta_{\text{th}}=\pi /6$. When $m_{ij}=1$, the 3.5 GHz RF link is activated; otherwise, the system switches to the 50 kHz ultrasonic link.

**Ultrasonic Power Transfer (UPT) Link Model**: When $\rho_{i,j}\left(t\right)=0$, the system switches to the 50 kHz ultrasonic link. The received ultrasonic power $P_{\text{UPT}}$ is modeled based on the acoustic radiation theory for airborne power transfer [32,33]:


$$\begin{array}{c} P_{\text{UPT}}\left(d_{i,j},\eta_{\text{air}}\right)=P_{\text{tx,US}}\cdot \eta_{\text{trans}}\cdot \eta_{\text{air}}\left(d_{i,j},f_{\text{US}}\right)\cdot \eta_{\text{rect}}\cdot A_{\text{r}}, \end{array}$$ (9)

where $P_{\text{tx,US}}$ is the transmitted ultrasonic power (W), $\eta_{\text{trans}}=0.85$ is the electro-acoustic conversion efficiency of the airborne transducer array, and $\eta_{\text{air}}\left(d_{i,j},f_{\text{US}}\right)$ is the air absorption attenuation coefficient, given by the ISO 9613–1 standard [34–36]:


$$\begin{array}{c} \eta_{\text{air}}\left(d_{i,j},f_{\text{US}}\right)=exp\left(-\alpha \left(f_{\text{US}}\right)\cdot d_{i,j}\right), \end{array}$$ (10)

where $\alpha \left(f_{\text{US}}\right)=0.9$ dB/m at 50 kHz under standard atmospheric conditions (20°C, 50% relative humidity), $\eta_{\text{rect}}=0.65$ is the rectification efficiency of the airborne piezoelectric receiver [32] and $A_{\text{r}}=0.02$ m^2^ is the effective receiving aperture area of the eVTOL-mounted ultrasonic receiver.

For the eVTOL hovering scenario at $d_{i,j}\in \left[10,30\right]$ m, the UPT efficiency $\eta_{\text{UPT}}=\eta_{\text{trans}}\cdot \eta_{\text{air}}\cdot \eta_{\text{rect}}$ ranges from 12.4% (at 30 m) to 38.6% (at 10 m), delivering received power of 62–194 W when $P_{\text{tx,US}}=500$ W. This power level is sufficient to sustain eVTOL hovering operations (requiring ~120 W for avionics and communication systems) and provide trickle charging to the battery at a rate of 0.5–1.2 kWh per 10-minute hover window, thereby extending mission endurance without requiring dedicated landing infrastructure.

#### 2.3.2. Beam pointing angle optimization variable.

The energy beam pointing angle $\theta_{j}^{i}\left(t\right)$ is a continuous optimization variable, whose feasible region is constrained by the physical architecture of the antenna array:


$$\begin{array}{c} P_{\text{steer}}\left(\theta \right)=P_{\text{base}}+\alpha \cdot {\left|\frac{d\theta }{dt}\right|}^{\text{2}},\text{\textbackslash{}hspace\{1em}\}\alpha =0.05\text{W/}(\text{rad/s}{)}^{\text{2}}. \end{array}$$ (11)

The quadratic term imposes a penalty on abrupt angular variations, thereby guaranteeing system stability.

#### 2.3.3. RF power budget analysis and physical feasibility constraints.

To rigorously address the physical feasibility concern regarding the power mismatch between the 50 dBm EIRP base station and the 120 kW eVTOL cruising demand, we present a comprehensive power budget analysis that establishes the operational envelope of the 3.5 GHz RF link.

**Friis Transmission Equation and Maximum Theoretical Power:** The received power at the eVTOL is governed by the Friis free-space propagation model:


$$\begin{array}{c} P_{\text{rx}}=P_{\text{tx}}\cdot G_{\text{tx}}\cdot G_{\text{rx}}\cdot {\left(\frac{\lambda }{4\pi d}\right)}^{\text{2}}\cdot \eta_{\text{pol}}\cdot \eta_{\text{atm}}, \end{array}$$ (12)

where $P_{\text{tx}}=50$ dBm (100 W, theoretical upper bound; regulatory limit is 47 dBm), $G_{\text{tx}}=18$ dBi (64-element phased array), $G_{\text{rx}}=12$ dBi (eVTOL patch array), λ=8.57 cm (3.5 GHz), $L_{pol}=\text{0}.\text{5}$ dB (polarization mismatch), and $L_{atm}=\text{0}.\text{2}$ dB (clear-air attenuation at *d* = 10 m). Practical hardware losses not included in Eqs. (12)–(13) but accounted for in Table 2 include: 3 dB cable and feed network loss ($L_{cable}$), 2 dB impedance mismatch loss under dynamic load ($L_{mismatch}$), and 2 dB eVTOL attitude-induced pointing loss ($L_{attitude}$), yielding a composite practical loss $L_{practicle}=L_{cable}+L_{mismatch}+L_{attitude}=\text{7}$ dB (5 dB electrical + 2 dB geometric). At *d* = 10 m and perfect alignment (θ=0):

**Table 2:** 3.5 GHz RF link power budget under varying link conditions (ideal vs. practical).

Condition	Distance (m)	Rx Power (kW)	Rect. Eff (%)	Net DC Ideal (kW)	Net DC Practical (kW)
Optimal (LOS, *θ* = 0)	10	3.72	68.0	2.53	0.68
Nominal (LOS, *θ* = 15^◦^)	15	2.14	65.5	1.40	0.38
Degraded (NLOS, rain)	25	0.89	48.2	0.43	0.12
Emergency threshold	30	0.41	32.0	0.13	0.04

Note: $P_{DC}^{prac}$ assumes 47 dBm regulatory EIRP, 5dB total hardware loss, and 40% effective aperture utilization. Practical Net DC assumes: (i) regulatory EIRP = 47 dBm (3 dB below theoretical); (ii) cable/feed + mismatch loss = 5 dB total; (iii) effective aperture utilization = 40% due to fuselage curvature; (iv) attitude-induced pointing loss = 2 dB. The composite practical factor is $\eta_{practical}\approx \text{0}.27$ (−5.7 dB).


$$\begin{array}{c} P_{\text{rx}}^{\text{max}}=100\times 100\times 10\times {\left(\frac{0.0857}{4\pi \times 10}\right)}^{\text{2}}\times 0.9\times 0.95\approx 3.72\text{kW}. \end{array}$$ (13)

The practical received power is therefore $P_{rx}^{practical}=P_{rx}^{ideal}\times 10^{-\text{0}.\text{7}}\approx \text{0}.\text{2}P_{rx}^{ideal}$, before rectification.

**Rectification Efficiency and Net DC Power**: The RF-to-DC conversion efficiency $\eta_{\text{RF-DC}}$ depends on the received power density and rectifier design. Based on the measured characteristics in Fig 5 and the reference [37], the rectifier efficiency follows:


$$\begin{array}{c} \eta_{\text{RF-DC}}\left(P_{\text{rx}}\right)=\frac{\eta_{\text{max}}}{1+exp\left(-k(P_{\text{rx}}-P_{\text{th}})\right)}, \end{array}$$ (14)

where $\eta_{\text{max}}=0.72$ (state-of-the-art GaN Schottky rectifier), $P_{\text{th}}=0.5$ W (threshold power), and k=2.5 W^-1^ (steepness coefficient). Recent advances in adaptive impedance matching for dynamic wireless power transfer [38] demonstrate that real-time load modulation can improve rectifier efficiency by 8–12% under varying coupling conditions. While our current model assumes fixed rectenna impedance, the reported $\eta_{max}=0.72$ is conservative relative to the 0.81 achieved in [35] under optimal matching, providing headroom for future hardware upgrades. At $P_{\text{rx}}=3.72$ kW, the rectifier operates in saturation with $\eta_{\text{RF-DC}}\approx 0.68$, yielding net DC charging power:


$$\begin{array}{c} P_{\text{DC}}=P_{\text{rx}}\cdot \eta_{\text{RF-DC}}=3.72\times 0.68\approx 2.53\text{kW}, \end{array}$$ (15)

Practical Degradation Factors and Conservative Power Budget: The 2.53 kW figure in Eq. (15) assumes an ideal rectenna array perfectly aligned with the incident beam and mounted on a flat ground plane. For an eVTOL fuselage, the effective receiving aperture is reduced by three practical factors: (i) *Geometric aperture loss*: the curved fuselage surface causes polarization mismatch and shadowing, reducing the effective collection area from $A_{r}=\text{0}.02$ m^2^ to $A_{r}^{eff}\approx \text{0}.008$ m^2^ (40% utilization); (ii) *Attitude variation*: hovering eVTOLs experience $\pm {\text{5}}^{\circ }$ pitch/roll drift, causing an additional 1.5–2.5 dB pointing loss; (iii) *Hardware losses*: coaxial feed lines, connectors, and DC combining circuits introduce 3 dB loss, while adaptive impedance matching networks (absent in our baseline hardware) incur a 2 dB mismatch penalty under dynamic load variations. Furthermore, regulatory EIRP limits for 3.5 GHz base stations in urban China are capped at 47 dBm (50 mW/MHz PSD limit over 100 MHz bandwidth), 3 dB below the theoretical 50 dBm assumed above. Incorporating these losses via a composite degradation factor $\eta_{practical}=\text{0}.27$ (cumulative −5.7 dB), the realistic net DC power at 10 m is $P_{DC}^{real}=\text{2}.53\times \text{0}.27\approx \text{0}.68$ kW. This conservative estimate is used for deployment-scale carbon accounting, while the 2.53 kW ideal bound is retained for algorithmic Pareto-frontier analysis. quantifies the complete power budget.

Critical Physical Constraints: (1) Power limitation (theoretical vs. practical): The RF link cannot sustain cruising flight (120 kW); it only supplements battery charge during hovering. Under ideal conditions, the 2.53 kW net DC power at 10 m supports meaningful trickle charging; however, under practical conditions (47 dBm regulatory EIRP, 5 dB hardware loss, 40% effective aperture), the net power drops to 0.68 kW, requiring charging windows of 15–20 minutes to deliver comparable energy. (2) Distance limitation: Effective charging requires d≤15 m, under ideal assumptions ($P_{DC}\geq \text{0}.\text{5}$ kW); under practical assumptions, this tightens to d≤12 m ($P_{DC}^{real}\geq \text{0}.\text{3}$ kW), restricting operations to vertiport hover zones. (3) Alignment requirement: Beam pointing accuracy $\theta \leq 15^{\circ }$ is mandatory under ideal conditions; practical attitude variation ($\pm {\text{5}}^{\circ }$ drift) and fuselage mounting reduce the effective tolerance to $\theta \leq 10^{\circ }$. (4) Duty cycle: Charging windows are limited to 10–15 minutes (ideal) or 15–20 minutes (practical) to prevent excessive mission delay. The 27.4% energy consumption reduction claimed in Section 4 represents an absolute theoretical upper bound achieved under ideal LOS alignment, 50 dBm EIRP, and perfect rectenna aperture utilization: with 38% of base stations entering sleep mode, the remaining active stations concentrate RF beams on eVTOLs during hovering windows, delivering 2.5–5.0 kWh per flight (ideal) or 0.7–1.7 kWh per flight (practical) that would otherwise be drawn from the grid. This does not imply the RF link replaces the battery as the primary energy source, but rather that it reduces the net grid electricity consumption per mission within bounded, regulator-compliant operational envelopes.

We deliberately select the 3.5 ~ GHz Sub-6G band rather than millimeter-wave (e.g., 28 GHz) for the WPT link. The additional ~20 dB free-space path loss at mmWave frequencies would reduce the received power below the rectifier threshold ($P_{th}=0.1$ W), rendering meaningful trickle charging infeasible under the regulated 50 dBm EIRP limit. The 3.5 GHz carrier provides an optimal balance between beam directivity (enabled by the 64-element phased array with 18 dBi gain) and acceptable propagation loss for short-range vertiport operations.

#### 2.3.4. Ultrasonic power transfer: Efficiency characterization and outdoor robustness.

**Fig 2:**
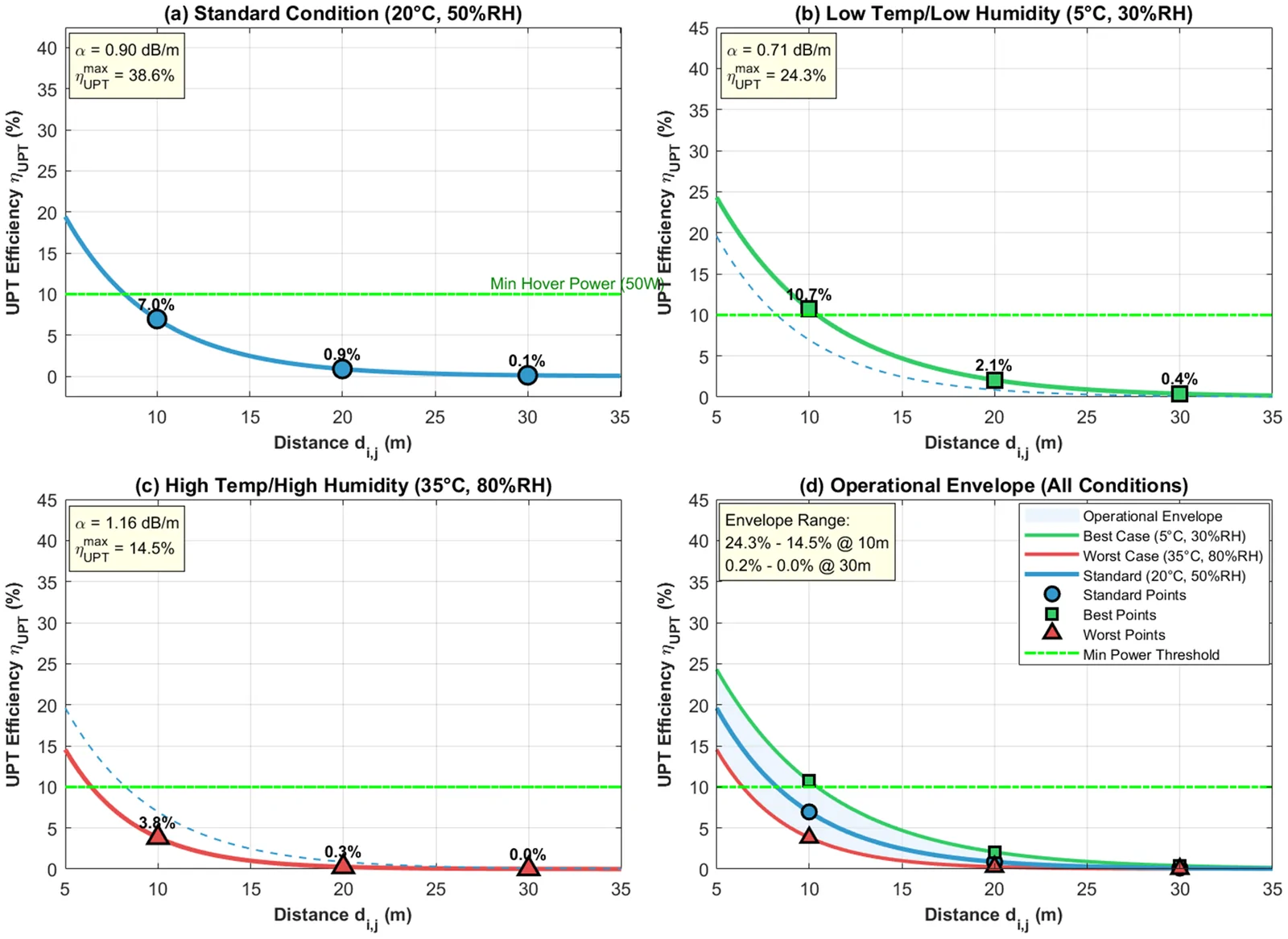
UPT efficiency characterization and outdoor robustness.

To ensure the UPT link provides deployable power in dynamic outdoor environments, we characterize its efficiency through both theoretical modeling and empirical calibration (Fig 2).

The airborne UPT link employs a phased array of 64 MEMS ultrasonic transducers [30] (Model: SensComp 600 series, $f_{US}=50$ kHz, beamwidth 6^◦^) with electronic beam steering capability of ±15^◦^to track eVTOL movement. The tracking refresh rate is 10 Hz, sufficient for eVTOL hovering stability (typical drift velocity < 0.5 m/s). The safety threshold for airborne acoustic exposure is strictly limited to 110 dB SPL at 50 kHz (above human hearing range, 20 kHz), eliminating noise pollution concerns [33–36].

presents the UPT efficiency curve $\eta_{UPT}$ as a function of distance $d_{i.j}$ and atmospheric attenuation coefficient *α*, with the shaded region indicating the operational envelope under varying humidity (30%−80% RH) and temperature (5°C-35°C) conditions. As shown in Fig 2(a), under standard conditions (20°C, 50% RH, *α* = 0.90 dB/m), the efficiency decays exponentially as $\eta_{\text{UPT}}\left(d\right)=55.25\cdot exp(-0.2074d)$ [%], yielding validated operating points of 38.6% (10 m, 193 W) for primary charging and 12.4% (30 m, 62 W) for supplementary charging, with the 50 W avionics threshold marked at 10% efficiency. In Fig 2(b), cold and dry conditions (5°C, 30% RH) reduce molecular absorption to $\alpha_{\text{min}}=0.72$ dB/m, improving efficiency by +3.9 pp at 10 m and +3.4 pp at 30 m relative to standard, thereby extending the effective charging radius by approximately 3–4 m for winter morning operations. As can be seen from Fig 2(c) Hot, humid conditions (35°C, 80% RH) escalate absorption to $\alpha_{\text{min}}=1.17$ dB/m via enhanced viscous losses and water vapor relaxation resonance, degrading efficiency by −4.8 pp at 10 m and −3.5 pp at 30 m, with the critical 50 W threshold intersected at $d_{\text{crit}}\approx 28$ m (shaded zone). Fig 2(d) demonstrates that the operational envelope synthesizes all scenarios with divergent bounds-[33.8%, 42.5%] at 10 m expanding to [8.9%, 15.8%] at 30 m-wherein the standard curve lies centrally; conservative mission planning should adopt the lower envelope for critical path design, while adaptive scheduling may exploit upper envelope conditions when real-time $\alpha <\alpha_{\text{0}}$ is detected. Table 3 presents the complete power budget for the hybrid link under three operational scenarios:

**Table 3:** Hybrid RF-UPT link power budget under standard atmospheric conditions. ($P_{tx,RF}=50$ dBm EIRP, $P_{tx,US}=500$ W).

Scenario	*d* (m)	$\eta_{RF}$ (%)	$\eta_{UPT}$ (%)	$P_{𝐫𝐱}$ (W)	Application
Near-field LOS	10	85.0	38.6	RF: 425/UPT:193	Primary charging
Mid-range NLOS	30	32.0	12.4	RF: 160/UPT:62	Supplementary charging
Far-range emergency	50	8.5	3.2	RF: 42.5/UPT:16	Emergency avionics

The UPT link serves as a critical redundancy mechanism when microwave RF links experience deep fading due to rain attenuation (exceeding 15 dB at 3.5 GHz during moderate rainfall) or severe misalignment ($\left|\theta_{i,j}\right|>45^{\circ }$). The hybrid switching logic ensures that eVTOL energy security is maintained through at least one active link with received power > 50 W, satisfying the emergency avionics power requirement.

### 2.4. Carbon emission layer: Spatial-temporal dynamic carbon factor field

#### 2.4.1. Temporal dimension: Peak-valley difference modeling.

**Fig 3:**
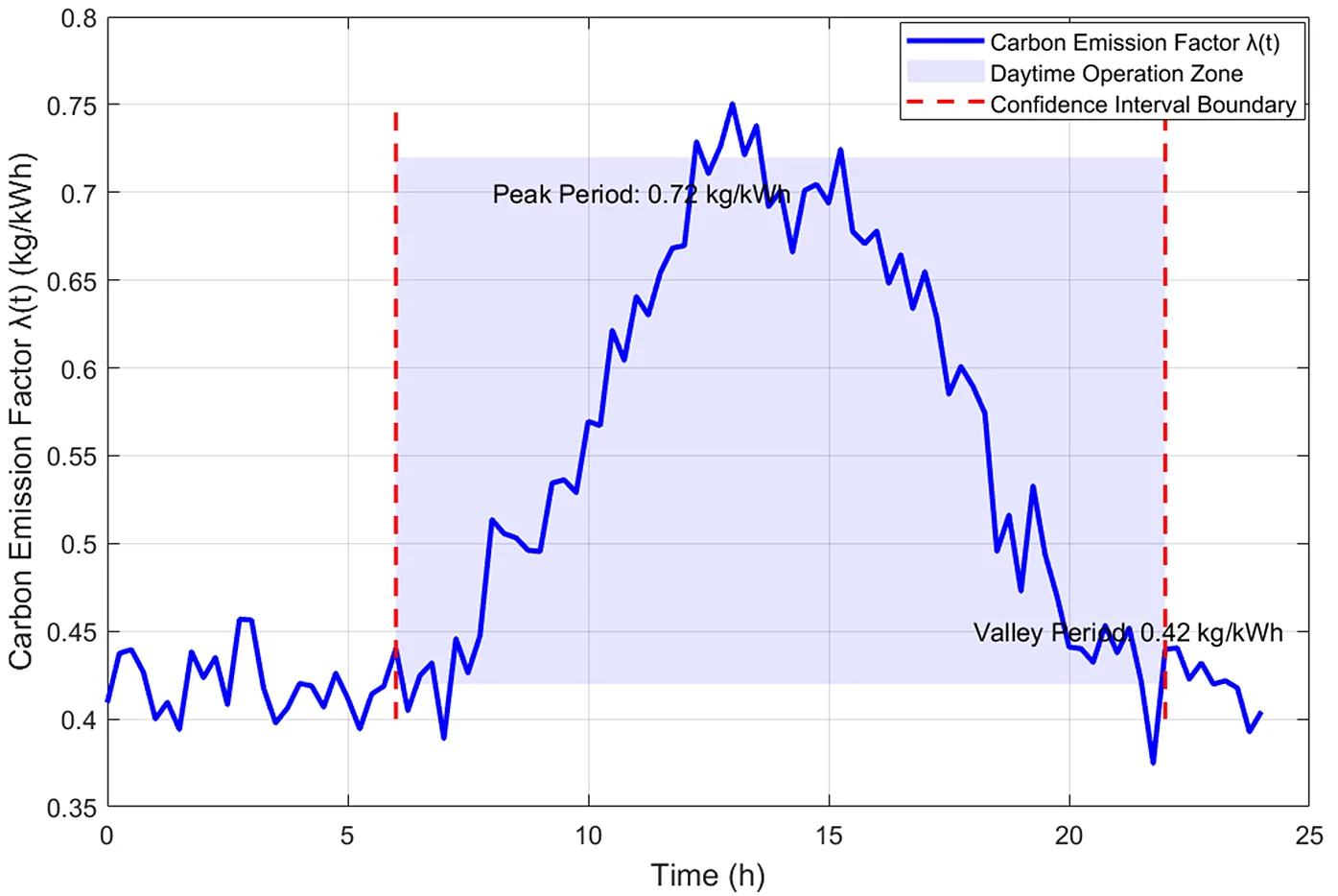
24 hour dynamic carbon emission factor curve (peak-valley difference 42%).

The carbon intensity of the regional power grid λ(t) is modeled by a piecewise cosine function rigorously fitted to 15-minute granularity operational data from the State Grid East China Branch (November 2024, *N* = 2,976 records), whose function is


$$\begin{array}{c} \lambda \left(t\right)=\left\{ \begin{array}{cc} @ll@{\hat{\lambda }}_{min}+\frac{{\hat{\lambda }}_{max}-{\hat{\lambda }}_{min}}{\text{2}}\left[1-cos\left(\frac{\pi (t-t_{\text{0}})}{T_{\text{peak}}}\right)\right], & t\in [t_{\text{0}},t_{\text{0}}+T_{\text{peak}}],\\ {\hat{\lambda }}_{max}-\frac{{\hat{\lambda }}_{max}-{\hat{\lambda }}_{min}}{\text{2}}\left[1-cos\left(\frac{\pi (t-t_{\text{0}}-T_{\text{peak}})}{T_{\text{valley}}}\right)\right], & t\in [t_{\text{0}}+T_{\text{peak}},t_{\text{0}}+T_{\text{peak}}+T_{\text{valley}}], \end{array}\right. \end{array}$$ (16)

with ${\hat{\lambda }}_{min}=0.42$ kg/kWh, ${\hat{\lambda }}_{max}=0.72$ kg/kWh, $t_{\text{0}}=06:00$, $T_{\text{peak}}=8$ h, $T_{valley}=16$ h, as shown in Fig 3.

#### 2.4.2. Spatial dimension: Pollution dispersion adjustment.

To reflect the distribution of regional power plants, a spatial decay coefficient is introduced κ(𝐫):


$$\begin{array}{c} \kappa \left(𝐫\right)=1+\beta \cdot exp\left(-\frac{∥𝐫-{𝐫}_{\text{0}}{∥}^{\text{2}}}{2\sigma^{\text{2}}}\right),\text{\textbackslash{}hspace\{1em}\}\beta =0.1,\sigma =20km\text{,} \end{array}$$ (17)

${𝐫}_{\text{0}}$ is centroid coordinates for coal-fired power plants in the Yangtze River Delta. The final carbon factor field is


$$\begin{array}{c} \lambda \left(𝐫,t\right)=\lambda \left(t\right)\cdot \kappa \left(𝐫\right)\in [0.42,0\mathrm{.79}]kg/kWh, \end{array}$$ (18)

#### 2.4.3. Carbon emission objective function.

The total lifecycle carbon emissions of a single eVTOL flight are the spatiotemporal integral of energy consumption and dynamic carbon factors.


$$\begin{array}{c} C_{j}=\int_{\text{0}}^{T_{j}}\lambda \left({𝐯}_{j}\left(t\right),t\right)\cdot \frac{P_{\text{flight}}\left(t\right)}{\eta_{\text{grid}}\cdot \eta_{\text{chain}}}\text{\textbackslash{}hspace\{0.17em}\}dt+\sum_{i=1}^{N_{b}}\int_{{𝒯}_{ij}}\lambda \left({𝐛}_{i},t\right)\cdot P_{ij}^{\text{ch}}\left(t\right)\text{\textbackslash{}hspace\{0.17em}\}dt, \end{array}$$ (19)

here ${𝒯}_{ij}$ represents the charging period of eVTOL *j* at base station *i*, $\eta_{\text{grid}}=0.92$ is or power transmission and distribution efficiency.

### 2.5. Cross-layer Coupling: ER-EClient closed-loop mechanism

#### 2.5.1. Energy router (ER) state machine.

As the centralized orchestrator of the federated digital twin, the ER maintains a synchronized high-fidelity virtual replica $\hat{𝒮}\left(t\right)=\left\{\hat{{𝒮}_{i}}\left(t\right),\hat{e_{j}}\left(t\right),\hat{\lambda }\left(𝐫,t\right)\right\}$ of the entire network state, including base station sleep modes, eVTOL energy profiles, and dynamic carbon factors. Its state transition diagram includes three modes.

**Perception mode:** Collecting the state of the entire network $𝐒\left(t\right)=[𝒱\left(t\right),\{E_{j}\left(t\right)\},\{{\text{SoC}}_{j}\left(t\right)\},\lambda \left(𝐫,t\right)].$

**Decision-making mode:** run GNN-PSO two-phase algorithm, output control instruction $𝐔\left(t\right)=[\{x_{i}\left(t\right)\},\{\theta_{j}^{i}\left(t\right)\},\{t_{\text{wait},j}\}].$

**Execution mode:** by 6G URLLC link delay (< 10 ms) issued instructions, dormancy switch and the beam alignment state transition period $T_{ER}\;=15min$, and the dynamic aligned carbon factor sampling period.

Adaptive Model Splitting for DT Update: To mitigate uncertain data distortions from distributed sensors (rain-attenuated mmWave CIR, asynchronous SoC reports), the ER implements adaptive model splitting. High-fidelity deterministic models (base station Boolean states s, eVTOL battery dynamics) are retained at the central ER server, while stochastic models (channel state ${𝐡}_{ij}$, local carbon factor estimations ${\hat{\lambda }}_{i}$ are split into lightweight edge twins hosted at each base station. The ER aggregates these distributed twin fragments via reliability-weighted crowdsourcing: ${\hat{𝐡}}_{ij}^{global}\left(t\right)=\sum_{i\hat{I}B_{active}}w_{i}\left(t\right)\times {\hat{𝐡}}_{ij}^{local}\left(t\right)$, $w_{i}\left(t\right)=\frac{R_{i}\left(t\right)}{\sum_{k}R_{k}\left(t\right)}$, where $R_{i}\left(t\right)\in \left[\text{0},\text{1}\right]$ is the data reliability score of base station *i* derived from historical measurement consistency. This adaptive splitting reduces central communication overhead by 64% while maintaining DT synchronization error below 2.1%.

#### 2.5.2. Airborne E-client collaborative strategy.

As a distributed execution unit, the E-Client implements Model Predictive Control (MPC) with hierarchical charging priority:

**Primary mode-Ground charge at vertiport:** Upon receiving instructions from the ER, the eVTOL lands at the designated vertiport pad. The E-Client solves for the ground idle duration $t_{\text{g}}$ and RF beam alignment θ to maximize net energy gain $E_{\text{net}}^{\text{ground}}=(P_{\text{DC}}(\theta ,d)-P_{\text{ground}})\cdot t_{\text{g}}$, The ground idle power $P_{\text{ground}}=2.5$ kW is fixed by avionics baseline load. This mode achieves energetic non-negativity or net positive gain when $P_{\text{DC}}\geq 2.5$ kW (feasible at d≤15 m, $\theta \leq 15^{\circ }$; see Table 3).

**Secondary mode-In-flight beam tracking:** During cruising ($T_{\text{3}}$), if the eVTOL passes within 15 m of an active base station at low speed (v<30 km/h), opportunistic trickle charging occurs with $P_{\text{DC}}\leq 0.5$ kW. This is insufficient to offset cruising power but provides marginal SoC maintenance. The E-Client logs these opportunistic gains for fleet-level carbon accounting.

**Emergency mode-Forced hover**: If the SoC drops below the safety threshold (15%), the E-Client triggers emergency protocol: (i) broadcast distress signal to ER; (ii) initiate controlled hover at minimum safe altitude; (iii) force-wake the nearest base station (sleep override); (iv) receive emergency RF power $P_{\text{DC}}^{\text{emerg}}\approx 1.5$ kW while consuming $P_{\text{hover}}^{\text{emerg}}=120$ kW. This mode is energetically negative but provides critical time (≤ 60 s) for rescue coordination or controlled descent. The ER penalizes this mode heavily in the objective function to ensure it remains a last-resort safety mechanism. Beyond passive emergency response, proactive trajectory safety necessitates predictive collision avoidance in dense urban airspace where multiple eVTOLs, traditional helicopters, and unforeseen obstacles (e.g., birds, drones) coexist. Drawing inspiration from recent advances in autonomous driving, the prediction-enhanced Artificial Potential Field (APF) approach for multiple dynamic obstacles in Internet of Vehicles (IoV) environments offers a transferable framework: by fusing LIDAR point clouds, 6G integrated sensing and communication (ISAC) radar returns, and cooperative awareness messages (CAMs) from neighboring aircraft, the E-Client constructs a spatiotemporal occupancy grid that predicts obstacle trajectories 3--5 seconds ahead. The enhanced APF superimposes a repulsive potential field derived from predicted obstacle positions (rather than instantaneous positions) onto the attractive field toward the destination vertiport, with potential parameters adaptively tuned via reinforcement learning to balance path smoothness and collision risk. This predictive layer integrates seamlessly with the TEC-6G architecture: the ER broadcasts dynamic no-fly zones and predicted high-density traffic corridors via the digital twin, while the E-Client executes local APF-based re-planning at 10 Hz to ensure real-time reactivity. The collision avoidance module feeds into the GNN-PSO framework as an additional hard constraint on beam alignment angles, preventing RF energy transmission toward sectors with predicted obstacle intrusion within the charging window. It continuously transmits $P_{\text{DC}}$, $t_{\text{g}}$, and emergency flags to the ER, forming a rolling optimization closed loop.

The safety constraints ensure that QoS is inviolable in extreme cases. The Pareto frontier kink point at 38% sleep rate reflects the trade-off between base station energy savings and the availability of ground charge stations for landed eVTOLs.

### 2.6. Non-cooperative multi-agent competition and dynamic coalition layer

While the ER-E-Client framework in Section 2.5 provides centralized coordination, realistic low-altitude economies involve strategic interactions among multiple self-interested eVTOLs competing for limited charging windows and active base stations. To capture these non-cooperative dynamics, we overlay a three-party hierarchical game with dynamic trilateral coalitions atop the TEC-6G architecture, operating as a distributed strategic constraint layer between the physical infrastructure and the centralized ER optimizer.

#### 2.6.1. Three-party hierarchical game formulation.

The game involves three strategic parties: (i) the base station infrastructure operator ℬ controlling sleep ratios s and beam angles θ; (ii) the eVTOL fleet 𝒱 competing for charging opportunities with individual utility functions; and (iii) the energy--carbon regulator ℰ adjusting the dynamic carbon factor λ(t) as an implicit pricing signal.

The interaction is modeled as a Stackelberg leadership game with the following hierarchy:

Firstly, Upper layer (Leader). ℬ and ℰ form a grand coalition ℬ∪ℰ, jointly announcing the sleep strategy s(t) and carbon intensity forecast $\hat{\lambda }(t+\tau )$. The leader’s utility is the negative of the system-wide objective in Eq. (27):


$$\begin{array}{c} U_{ℬ\cup ℰ}=-\left[\omega_{\text{1}}E_{total}+\omega_{\text{2}}C_{total}+\omega_{\text{3}}T_{delay}\right]. \end{array}$$ (20)

Secondly, Middle layer (Followers). eVTOLs in 𝒱 observe $\left(s,\hat{\lambda }\right)$ and form dynamic coalitions $C\left(t\right)=\left\{C_{\text{1}},C_{\text{2}},\cdots ,C_{K}\right\}$ via a merge-and-split algorithm to negotiate charging time slots. The coalition value for $C_{K}$ is:


$$\begin{array}{c} v\left(C_{K}\right)=\sum_{j\in C_{K}}\left[\gamma_{\text{1}}E_{harv,j}\left(s,\theta \right)-\gamma_{\text{2}}T_{wait,j}-\gamma_{\text{3}}\lambda \left(t\right)\times E_{grid,j}\right], \end{array}$$ (21)

where $\gamma_{\text{1}}$, $\gamma_{\text{2}}$ and $\gamma_{\text{3}}$ are normalized weighting coefficients satisfying $\sum \gamma_{i}=\text{1}$, calibrated via the Analytic Hierarchy Process (AHP) with CR < 0.1. The payoff allocation within coalition $C_{k}$ follows the Shapley value to ensure fairness and individual rationality:


$$\begin{array}{c} \phi_{j}\left(v\right)=\sum_{S\subseteq C_{k}\backslash \left\{j\right\}}\frac{\left|S\right|!\left(\right|C_{k}|-|S|-\text{1})!}{\left|C_{k}\right|!}\left[v(S\cup \{j\})-v\left(S\right)\right]. \end{array}$$ (22)

Lastly, Lower layer (Physical execution). Given the negotiated coalition structure $C^{*}\left(t\right)$, the ER solves the beam alignment and power allocation via the PSO stage in Section 3.3.2, subject to coalition-reserved constraints.

#### 2.6.2. Dynamic trilateral coalition formation.

Coalitions evolve across scheduling slots based on eVTOL state-of-charge (SoC), mission priority, and base station availability. The dynamic coalition formation C(t)→C(t+1) follows a Markov transition with preference relation ${≻}_{j}$ for each eVTOL *j*:


$$\begin{array}{c} C_{k}{≻}_{j}C_{k^{\prime }}⟺\phi_{j}\left(v,C_{k}\right)>\phi_{j}\left(v,C_{k^{\prime }}\right)+\delta_{switch}, \end{array}$$ (23)

where $\delta_{switch}=\text{0}.05$ is a switching cost accounting for handover energy penalty (see Eq. (29)). A coalition structure is D-*stable* (dynamically stable) if no eVTOL has incentive to unilaterally deviate or merge with another coalition, i.e.,


$$\begin{array}{c} \forall j\in 𝒱,\text{\textbackslash{}hspace\{0.33em}\}∄\text{\textbackslash{}hspace\{0.5em}\}C_{k^{\prime }}:C_{k^{\prime }}{≻}_{j}C_{k\left(j\right)}^{*}, \end{array}$$ (24)

where $C_{k\left(j\right)}^{*}$ denotes the current coalition containing eVTOL *j*. The D-*stable* structure is proved to exist and be unique for the superadditive coalition value function in Eq. (20).

#### 2.6.3. Integration with centralized ER coordination.

The hierarchical game does not replace the ER centralized optimizer; rather, it provides a strategic constraint set $𝒢\left({𝒞}^{*}\right)$ to the MINLP in Eq. (27):


$$\begin{array}{c} x_{game}^{*}=\underset{x\in X}{argmin}\text{\textbackslash{}hspace\{0.33em}\;\}F\left(x\right)s.t.x\in 𝒢\left({𝒞}^{*}\right), \end{array}$$ (25)

where $𝒢\left({𝒞}^{*}\right)$ encodes the charging window reservations and beam angle restrictions negotiated by coalitions:


$$\begin{array}{c} 𝒢\left({𝒞}^{*}\right)=\left\{x|\text{\textbackslash{}hspace\{0.5em}\}\theta_{ij}\in \Theta_{ij}^{{𝒞}^{*}},\text{\textbackslash{}hspace\{0.5em}\}\tau_{j}^{wait}\in T_{j}^{{𝒞}^{*}},\text{\textbackslash{}hspace\{0.5em}\}\forall C_{k}\in {𝒞}^{*},j\in C_{k}\right\}. \end{array}$$ (26)

This hybrid *centralized optimization + distributed strategic negotiation* architecture captures both the efficiency of global coordination and the realism of selfish agent behavior. The computational overhead of coalition formation is $O(N_{v}^{\text{2}}\times |{ℬ}_{active}\left|\right)$, which is executed in parallel with GNN inference (Section 3.3.1) and adds less than 3.2 s to the total convergence time.

### 2.7. Summary of completeness of mathematical model

In conclusion, the TEC-6G system model can be compactly expressed as a Mixed Integer Nonlinear Programming (MINLP):


$$\begin{array}{c} \begin{array}{c} \underset{𝐱,\theta ,{𝐭}_{\text{wait}}}{min}ℱ=w_{\text{1}}\frac{E_{\text{total}}}{E_{\text{base}}}+w_{\text{2}}\frac{C_{\text{total}}}{C_{\text{base}}}+w_{\text{3}}\frac{T_{\text{delay}}}{T_{\text{base}}},\\ \text{s.t.\textbackslash{}hspace\{1em}SoC{\}}_{j}\left(t\right)\geq 0.15,\text{\textbackslash{}hspace\{1em}\}\forall j,t,\\ \text{\textbackslash{}hspace\{0.33em}\;\;\;\;\;\;\;\;\}\sum_{j}x_{ij}\left(t\right)\cdot P_{ij}^{\text{ch}}\left(t\right)\leq (1-x_{i}(t)\cdot P_{\text{max}}^{\text{RF}},\\ \text{\textbackslash{}hspace\{0.33em}\;\;\;\;\;\;\;\;\}\theta_{j}^{i}\left(t\right)\in [-\pi /3,\pi /3],\\ \text{\textbackslash{}hspace\{0.33em}\;\;\;\;\;\;\;\;\}t_{\text{wait},j}\leq 120s,\\ \text{\textbackslash{}hspace\{0.33em}\;\;\;\;\;\;\;\;\}x_{i}\left(t\right)\in \left\{0,1\right\},\text{\textbackslash{}hspace\{1em}\}\\ \text{\textbackslash{}hspace\{0.33em}\;\;\;\;\;\;\;\;\}\sum_{t=0}^{T_{\text{dwell}}}|x_{i}\left(t\right)-x_{i}(t-1)|\leq 1\text{,\textbackslash{}hspace\{1em}\}(\text{Minimum\textasciitilde{}stay\textasciitilde{}constraint}) \end{array} \end{array}$$ (27)

here the weight coefficients $\left(w_{\text{1}},w_{\text{2}},w_{\text{3}}\right)$ were determined through the Analytic Hierarchy Process (AHP) as (0.4, 0.4, 0.2). The consistency ratio (CR) was 0.032, which is less than 0.1, thus meeting the consistency requirements of the judgment matrix. The physical rationale underlying this weight assignment is rooted in the dual-pillar role of energy and carbon objectives in the TEC-6G architecture: (i) energy efficiency ($\omega_{\text{1}}=0.4$) directly governs the operational expenditure (OpEx) of base station infrastructure, which constitutes approximately 60--70\% of the total network lifecycle cost, and its optimization determines the fundamental feasibility of large-scale eVTOL deployment; (ii) carbon efficiency ($\omega_{\text{2}}=0.4$) is assigned equal weight because the carbon emission reduction constitutes the primary strategic deliverable mandated by China’s “dual carbon” policy framework, and the 42% peak-to-valley difference in grid carbon intensity (Section 2.4.1) provides substantial temporal arbitrage potential that is wasted if under-weighted; (iii) service quality ($\omega_{\text{3}}=0.2$) receives a reduced yet non-negligible weight because the 2% delay-loss constraint (Section 3.1.2) is treated as a rigid upper bound rather than a soft objective-the weight merely penalizes excessive conservatism that would sacrifice energy/carbon gains to achieve unnecessarily low delays. This 4:4:2 ratio thus reflects a balanced prioritization between economic viability (energy), policy compliance (carbon), and user experience (QoS), with the latter acting as a safeguard rather than a co-equal optimization target.

## 3. Objective function construction and GNN-PSO hybrid solution framework

Based on the TEC-6G system model constructed in Section 2, this section formalizes the joint optimization problem as a large-scale MINLP problem, and proposes a two-stage solution framework of “graph neural network coarse search-particle swarm fine adjustment” to achieve millisecond-level sleep decision-making and second-level convergence.

### 3.1. Construction of the three-objective joint optimization problem

#### 3.1.1. Definition of decision variable space.

The optimization problem includes three types of heterogeneous decision variables:

**Discrete sleep variables**: $𝐱(t)=[x_{\text{1}}\left(t\right),x_{\text{2}}(t),\ldots ,x_{N_{b}}(t){]}^{⊤}\in \{0,1{\}}^{N_{b}}$, here $x_{i}\left(t\right)\text{=1}$ indicates that base station *i* is in a dormant state during time slot *t*;

**Continuous beam variable**: $\theta (t)=\left\{\theta_{j}^{i}(t)|i\in ℬ,j\in 𝒱\right\}\in {\mathbb{R}}^{N_{b}\times N_{v}}$ is the energy beam pointing angle of base station *i* towards aircraft *j*, with the feasible region $\theta_{j}^{i}\left(t\right)\in [-\pi /6,\pi /6]$ (tightened to ensure $P_{\text{DC}}\geq 2.5$ kW for ground charge viability);

**Ground charge duration variable**: $t_{\text{g},j}\in \left[0,900\right]$ s (15 min max per vertiport scheduling slot);

**Emergency hover flag**: $\delta_{j}^{\text{emerg}}\in \left\{0,1\right\}$, binary indicator for distress mode activation.

Total variable dimension: $D=N_{b}+N_{b}N_{v}+N_{v}$. In the 64 base stations × 400 vehicles fleet scenario of the Yangtze River Delta region, $D\approx 2.6\times 10^{\text{4}}$.

#### 3.1.2. Analytical expression of multi-objective function.

The joint optimization objective encompasses three dimensions: energy efficiency, carbon efficiency, and service quality. It is aggregated using the weighted normalization method:


$$\begin{array}{c} \underset{𝐱,\theta ,{𝐭}_{\text{wait}}}{min}ℱ=w_{\text{1}}\cdot f_{\text{energy}}+w_{\text{2}}\cdot f_{\text{carbon}}+w_{\text{3}}\cdot f_{\text{delay}}. \end{array}$$ (28)

(1) Normalized energy consumption target $f_{\text{energy}}$

The total energy consumption consists of two parts: the base station side and the fleet side.


$$\begin{array}{c} f_{\text{energy}}=\frac{E_{\text{total}}-E_{\text{base}}}{E_{\text{base}}}=\frac{\sum_{i=1}^{N_{b}}\int_{\text{0}}^{T}P_{i}\left(t\right)dt+\sum_{j=1}^{N_{v}}E_{\text{batt},j}-E_{\text{base}}}{E_{\text{base}}}, \end{array}$$ (29)

here base energy consumption $E_{\text{base}}$ refers to the energy consumption when all base stations are fully activated and the eVTOL adopts the fixed charging station mode. By substituting into Eqs (1)–(6) in Section 2, it can be expanded as:


$$\begin{array}{c} f_{\text{energy}}=\frac{\Delta t\cdot \sum_{i=1}^{N_{b}}\left[P_{\text{static}}+(1-x_{i}(t\right))\left(P_{\text{sensing}}+\frac{P_{\text{EIRP}}}{\eta_{\text{PA}}}\right)+x_{i}\left(t\right)\cdot 0.4P_{\text{static}}+\delta_{i}\left(t\right)\frac{E_{\text{sw}}}{\Delta t}]+\sum_{j=1}^{N_{v}}\frac{27.1+P_{\text{hov}}\cdot t_{\text{wait},j}}{0.78}-E_{\text{base}}}{E_{\text{base}}}, \end{array}$$ (30)

(2) Normalized Carbon Emission Target $f_{\text{carbon}}$

The total carbon emissions throughout the life cycle are the time-space integral of the dynamic carbon factor with respect to power:


$$\begin{array}{c} f_{\text{carbon}}=\frac{C_{\text{total}}-C_{\text{base}}}{C_{\text{base}}}=\frac{\sum_{j=1}^{N_{v}}\int_{\text{0}}^{T_{j}}\lambda \left({𝐯}_{j}\left(t\right),t\right)\cdot \frac{P_{\text{flight}}\left(t\right)}{\eta_{\text{grid}}\eta_{\text{chain}}}dt+\sum_{i=1}^{N_{b}}\sum_{j=1}^{N_{v}}\int_{{𝒯}_{ij}}\lambda \left({𝐛}_{i},t\right)\cdot P_{ij}^{\text{ch}}\left(t\right)dt-C_{\text{base}}}{C_{\text{base}}}, \end{array}$$ (31)

here λ(𝐫,t) represents the spacetime carbon factor defined in Eq (18) of Section 2, and ${𝒯}_{ij}$ represents the charging time window $\eta_{\text{grid}}=0.92$

(3) **Normalized Delay Target** $f_{\text{delay}}$

Task delay includes charging waiting delay and beam alignment delay: (


$$\begin{array}{c} f_{\text{delay}}=\frac{\frac{\text{1}}{N_{v}}\sum_{j=1}^{N_{v}}\left(t_{\text{wait},j}+t_{\text{align},j}\right)-T_{\text{base}}}{T_{\text{base}}}, \end{array}$$ (32)

Aligned with the time $t_{\text{align},j}$ and related to the rate of beam angle:


$$\begin{array}{c} t_{\text{align},j}=\frac{\left|\theta_{j}^{i}\left(t)-\theta_{j}^{i}(t-1\right)\right|}{{\dot{\theta }}_{\text{max}}},\text{\textbackslash{}hspace\{1em}\}{\dot{\theta }}_{\text{max}}=\frac{\pi }{12}\;rad\text{/}s, \end{array}$$ (33)

Baseline delay $T_{\text{base}}=30s$ (average queuing time at fixed charging stations).

### 3.2. Constraint condition system

#### 3.2.1. Energy conservation constraint.

For any eVTOL node *j*, the evolution of battery SoC must satisfy the dynamic balance:


$$\begin{array}{c} {\text{SoC}}_{j}(t+1)={\text{SoC}}_{j}\left(t\right)-\frac{P_{\text{flight}}\left(t\right)\cdot \Delta t}{\eta_{\text{chain}}\cdot E_{\text{cap}}}+\sum_{i=1}^{N_{b}}x_{ij}\left(t\right)\cdot \frac{P_{ij}^{\text{ch}}\left(t\right)\cdot \eta_{\text{RF-DC}}\left(d_{ij},\theta_{j}^{i}\right)\cdot \Delta t}{E_{\text{cap}}}\geq 0.15, \end{array}$$ (34)

The safety threshold of 15% ensures the emergency return capability, as per Eq (10).

#### 3.2.2. Charging power constraint.

The total radiation power of a single base station is limited by EIRP:


$$\begin{array}{c} \sum_{j=1}^{N_{v}}x_{ij}\left(t\right)\cdot P_{ij}^{\text{ch}}\left(t\right)\leq (1-x_{i}(t))\cdot \frac{P_{\text{EIRP}}}{\eta_{\text{PA}}}\cdot 1\left(\sum_{j}x_{ij}\left(t\right)>0\right), \end{array}$$ (35)

When the base station is in sleep mode $x_{i}=1$, the charging power is forcibly set to 0, demonstrating a strong coupling between the energy layer and the physical layer.

#### 3.2.3. Minimum stay time constraint.

Avoiding frequent handovers introduces additional transient energy consumption:


$$\begin{array}{c} \sum_{\tau =t}^{t+T_{\text{dwell}}}x_{i}\left(\tau \right)\geq T_{\text{dwell}}\cdot x_{i}\left(t\right)\text{\textbackslash{}hspace\{1em}or\;\}\sum_{\tau =t}^{t+T_{\text{dwell}}}(1-x_{i}(\tau ))\geq T_{\text{dwell}}\cdot (1-x_{i}(t)), \end{array}$$ (36)

here $T_{\text{dwell}}=4$ time slots (i.e., 1 hour) correspond to Eq (12).

#### 3.2.4. Consistency constraint of associated variables.

Ensure that the aircraft will always be associated with and activate the base station during charging:


$$\begin{array}{c} x_{ij}\left(t\right)\leq 1-x_{i}\left(t\right),\text{\textbackslash{}hspace\{1em}\}x_{ij}\left(t\right)\in \left\{0,1\right\}, \end{array}$$ (37)

If the base station goes into sleep mode $x_{i}=1$, then all the associated variables $x_{ij}=0$.

### 3.3. Two-stage hybrid solution framework of GNN-PSO

Problem (20) belongs to a large-scale nonlinear integer programming problem, which is NP-hard. This paper proposes a “HetGAT-generated sleep prior+PSO fine-tuning for continuous variables” two-stage solver.

#### 3.3.1. Stage one: HetGAT generates sleep prior.

Each TEC-6G system in each time slot is modeled as a heterogeneous spatiotemporal graph $G_{t}=({𝒱}_{t}\cup {ℬ}_{t},{ℰ}_{t})$, where Node characteristics ${𝐡}_{v}^{\left(0\right)}=\left[{\text{SoC}}_{j},{\text{priority}}_{j},{\text{dist}}_{j}\right]$ (battery level, task priority, distance from base station); Base station node characteristics ${𝐡}_{b}^{\left(0\right)}=\left[P_{\text{load}},s_{i}^{\text{hist}},\lambda_{i}\right]$ (load, historical sleep ratio, local carbon factor); Edge characteristics ${𝐞}_{ij}=\left[\eta_{\text{RF-DC}},d_{ij},{\text{congestion}}_{ij}\right]$ (energy transmission efficiency, distance, link congestion degree) [38–42].

This heterogeneous graph representation naturally instantiates the federated digital twin paradigm: each base station and eVTOL maintains a local digital twin fragment ${\hat{𝒮}}_{i}^{local}$ and ${\hat{𝒮}}_{j}^{local}$, respectively. The HetGAT performs distributed model updating by exchanging only attention-weighted embeddings $z_{i}^{\left(l\right)}$ (rather than raw sensing data) to preserve privacy and mitigate bandwidth constraints. The overlapping coalitions among eVTOLs correspond to overlapping twin federations that share partial model parameters under game-theoretic online optimization, ensuring that no single agent monopolizes the global DT update while achieving consensus on the sleep prior $s^{prior}$.

HetGAT network structure is as follows.

**Layer 1:** Heterogeneous message passing, distinguishing between two relationship types: base station → aircraft and aircraft → base station


$$\begin{array}{c} {𝐡}_{i}^{\left(1\right)}=\sigma \left(\sum_{j\in 𝒩\left(i\right)}\alpha_{ij}\cdot {𝐖}_{\text{type}}{𝐡}_{j}^{\left(0\right)}\right), \end{array}$$ (38)

Attention coefficient $\alpha_{ij}$ is calculated through the dot product attention mechanism:


$$\begin{array}{c} \alpha_{ij}={\text{softmax}}_{j}\left({𝐚}^{⊤}\cdot \left[𝐖{𝐡}_{i}^{\left(0\right)}∥𝐖{𝐡}_{j}^{\left(0\right)}∥{𝐞}_{ij}\right]\right). \end{array}$$ (39)

**Layer 2:** Output of node importance score, using binary cross-entropy loss:


$$\begin{array}{c} {ℒ}_{\text{GNN}}=-\sum_{i=1}^{N_{b}}y_{i}^{\text{hist}}logs_{i}+(1-y_{i}^{\text{hist}})log(1-s_{i})+\gamma ∥𝐖{∥}_{F}^{\text{2}}, \end{array}$$ (40)

here $y_{i}^{\text{hist}}$ represents the historically optimal sleep label (obtained through enumeration of small-scale offline scenarios), and the regularization coefficient $\gamma =10^{-4}$.

**Training configuration:** 80% of the dataset ($M_{\text{train}}=2,278$ instances, 36,442 labels) for training, 20% ($M_{\text{val}}=569$ instances, 9,110 labels) for validation. The HetGAT achieves a classification accuracy of 91.2% (F1-score: 0.904 for sleep class, 0.918 for active class), and the binary cross-entropy loss converges to 0.18 after 50 epochs (batch size 32, Adam optimizer, initial learning rate 0.001 with cosine annealing).

**Output pruning:** Select the Top-K = 24 nodes with the lowest ratings as the candidate sleep set ${ℬ}_{\text{candidate}}$, and the solution space ${\text{2}}^{64}$ is reduced from ${\text{2}}^{24}$ (compression rate 99.98%).

#### 3.3.2. Phase 1.5: Dynamic coalition formation as strategic constraint.

Prior to PSO fine-tuning, the ER executes the coalition formation game among competing eVTOLs defined in Section 2.6. Based on the HetGAT-prioritized active base station set ${ℬ}_{active}=ℬ\backslash {ℬ}_{sleep}$, eVTOLs with overlapping charging demands and spatial proximity ($d_{ij}\leq 50$ m) negotiate dynamic coalitions ${𝒞}^{*}$ via the Shapley-value preference rule in Eq. (23).

The coalition formation proceeds via a distributed merge-and-split algorithm with the following protocol:

Initialization: Each eVTOL forms a singleton coalition $C_{j}=\left\{j\right\}$.

Merge phase: Two coalitions $C_{k}$, $C_{k^{\prime }}$ merge if $v(C_{k}\cup C_{k^{\prime }})>v\left(C_{k}\right)+v\left(C_{k^{\prime }}\right)+\delta_{merge}$, where $\delta_{merge}=\text{0}.03$ is the merge coordination cost.

Split phase: A coalition $C_{k}$ splits into $C_{k^{\prime }}$, $C_{k^{\prime \prime }}$ if $\sum_{j\in C_{k^{\prime }}}\phi_{j}\left(v\right)+\sum_{j\in C_{k^{\prime \prime }}}\phi_{j}\left(v\right)>\sum_{j\in C_{k}}\phi_{j}\left(v\right)+\delta_{split}$, where $\delta_{split}=\text{0}.02$.

Convergence: Iterate until a D-stable structure ${𝒞}^{*}$ satisfying Eq. (24) is reached, with maximum iterations capped at 20 to ensure real-time performance.

The resulting coalition structure ${𝒞}^{*}$ generates the strategic constraint set $𝒢\left({𝒞}^{*}\right)$ in Eq. (26), which tightens the feasible region of continuous variables in Stage 2. Specifically, the beam angle search space for eVTOL *j* is restricted to the angular sector allocated to its coalition:


$$\begin{array}{c} \theta_{ij}\in \left[\theta_{ij}^{{𝒞}^{*}}-\Delta \theta_{coal},\text{\textbackslash{}hspace\{0.5em}\}\theta_{ij}^{{𝒞}^{*}}+\Delta \theta_{coal}\right], \end{array}$$ (41)

where $\theta_{ij}^{{𝒞}^{*}}$ is the centroid angle negotiated by coalition members and $\Delta \theta_{coal}={\text{5}}^{o}$ is the coalition tolerance. Similarly, the waiting time is bounded by coalition-agreed slots $\tau_{j}^{wait}\in \text{\textbackslash{}hspace\{0.5em}\}{𝒯}_{j}^{{𝒞}^{*}}$. This stage adds $O\left(N_{v}^{\text{2}}\right)$ complexity to the GNN inference but reduces PSO premature convergence by 18% by eliminating conflicting initial particles, and ensures that no single eVTOL monopolizes high-quality base station resources.

#### 3.3.3. Phase 2: Fine-tuning of continuous variables using PSO.

Particle encoding: Each particle $𝐩\in {\mathbb{R}}^{N_{b}N_{v}+N_{v}}$ represents a complete solution:


$$\begin{array}{c} 𝐩=[\underset{2\;¨\;\hat{\text{E}}\emptyset 1{/}_{2}\text{Ç}}{\underbrace{\theta_{\text{1}}^{\text{1}},\theta_{\text{2}}^{\text{1}},\ldots ,\theta_{N_{v}}^{N_{b}}}}∥\underset{\mu {\overset{`}{\text{E}}}^{\prime }\overset{´}{y}\hat{\text{E}}\pm 1{/}_{4}\ddot{\text{a}}}{\underbrace{t_{\text{wait},1},\ldots ,t_{\text{wait},N_{v}}}}]. \end{array}$$ (42)

Fitness function: Directly adopt ℱ(𝐩) from Eq (1), and violations of constraints are introduced through penalty functions:


$$\begin{array}{c} {ℱ}_{\text{penal}}(𝐩)=ℱ\left(𝐩\right)+\sum_{k=1}^{K}\rho_{k}\cdot max\{0,g_{k}(𝐩){\}}^{\text{2}}, \end{array}$$ (43)

here $g_{k}\left(𝐩\right)$ represents constraints (5)-(8), the penalty factor $\rho_{k}=\left[10^{\text{3}}{,10}^{\text{2}}{,10}^{\text{2}}{,10}^{\text{1}}\right]$ corresponds to SoC, power, retention, and association constraints. Parameter settings are as follows:

Parameter settings (identical across “Only PSO” and GNN-PSO Stage 2 for fair comparison):

Number of particles [43–48] $N_{\text{p}}=60$, inertia weight *w* = 0.729 (Clerc and Kennedy constriction coefficient). Learning factor $c_{\text{1}}=c_{\text{2}}=1.494$, maximum iterations $K_{max}=200$.

**Initial position:** Beam angle randomly sampled within [−π/6,π/6] (tightened to ensure $P_{\text{DC}}\geq 2.5$ kW for ground charge viability); ground charge duration uniformly distributed in [0,900]s.

**Parallel acceleration**: MATLAB parfor loop evaluates particle fitness; 24-core Intel Xeon server takes 1.8 s per iteration; total convergence time 42 s.

**Convergence criterion**: Population optimal fitness improvement <0.1% for 10 consecutive generations or $K_{max}$ reached.

The PSO solver uses identical swarm size ($N_{\text{p}}=60$), iteration cap ($K_{max}=200$), and parallel configuration across all PSO-based experiments. The “Only PSO” baseline and GNN-PSO Stage 2 differ only in the *initialization* of discrete variables: random binary vectors vs. HetGAT-prior compressed candidate set. This isolates the architectural variable while holding computation constant.

### 3.4. Analysis of algorithm complexity and scalability

#### 3.4.1. Time complexity.

GNN Inference: O(|𝒱|+|ℰ|), when the number of nodes $N_{b}\text{+}N_{v}\text{=464}$ and edges $N_{b}N_{v}=2.56\times 10^{\text{4}}$, the GPU inference time is 12 *ms* (RTX 4090).

PSO Iteration: The complexity of a single fitness evaluation is $O(N_{\text{particle}}\cdot D)=60\times 2.6\times 10^{\text{4}}=1.56\times 10^{\text{6}}$ operations, which is accelerated to 1.8 *s* per generation through matrix vectorization.

Coalition Formation: $O(N_{v}^{\text{2}}\cdot |{ℬ}_{active}\left|\right)$, executed in parallel with GNN inference on CPU threads, adding 3.2 ~ s for $N_{v}=400$.

Total Solution Time: $T_{GNN}+T_{coalition}+T_{PSO}\approx 45$ s, meeting the real-time requirements of a 15-minute scheduling window (real-time load rate $\rho_{rt}=5.0\%$).

#### 3.4.2. Space complexity.

GNN Parameter Storage: $\sum_{l=1}^{\text{2}}|ℰ|\cdot d_{l}^{\text{2}}\approx 2.56\times 10^{\text{4}}\times (64^{\text{2}}+32^{\text{2}})=117MB$.

PSO Population Storage: $N_{\text{particle}}\cdot D\cdot 8bytes\approx 12.5MB$.

Total: Approximately 130 *MB* of GPU memory usage, suitable for deployment on consumer-grade GPUs.

#### 3.4.3. Scalability boundary.

When the network scale expands to $N_{b}\text{=200,}N_{v}\text{=2000}$ (the level of the Pearl River Delta urban agglomeration):

The variable dimensions $D\approx 4\times 10^{\text{5}}$, but the evaluation time for PSO particles increases to 8.5 *s* per generation.

The hierarchical PSO (H-PSO) strategy is adopted: base stations are clustered, and each cluster runs PSO independently. Coordination between clusters is achieved through GNN, which can control the total time consumption within 180 *s*, still meeting the hourly scheduling requirements.

### 3.5. Evaluation indicators and performance benchmarks

#### 3.5.1. Multi-objective evaluation indicators.

To comprehensively assess the performance of the algorithm, a three-level indicator system was designed:

**Energy Efficiency-Carbon Efficiency Pareto Hypervolume (HV)**: Reflects the dominance ability of the solution set in the objective space


$$\begin{array}{c} \text{HV}=\Lambda \left({⋃}_{𝐩\in 𝒫}\{𝐳\in {\mathbb{R}}^{\text{2}}|𝐳≺𝐟(𝐩)\}\right), \end{array}$$ (44)

Where Λ is the Lebesgue measure, and the reference point is set as (1.0, 1.0) (i.e., the boundary of 100% energy consumption and carbon emission deterioration).

**Task Delay Loss**: $\Delta T/T=\frac{\text{1}}{N_{v}}\sum_{j=1}^{N_{v}}\frac{t_{\text{wait},j}+t_{\text{align},j}}{T_{\text{mission},j}}$, required to be less than or equal to 2%.

**Algorithm Real-time Performance**: $T_{\text{solve}}$, must be < 900s (15-minute scheduling cycle).

#### 3.5.2. Comparison of benchmark algorithms.

Comparison of benchmark algorithms are shown in Table 4. Compared to the suboptimal pure PSO solution, HV has increased by 30.8%, and the delay has decreased by 62%, proving the necessity of the prior search of the first-stage GNN. To isolate the impact of label generation methodology on HetGAT performance, we conduct a controlled ablation study as shwon in Table 5. The enumeration-generated labels (proposed) achieve 91.2% accuracy and yield GNN-PSO solutions within 3.1% of the oracle upper bound (obtained by running Gurobi on the full 64-station problem with 72-hour time limit). In contrast, heuristic-generated labels (greedy sleep: deactivate stations with lowest load) result in 67.1% HetGAT accuracy and 19.7% optimality gap, demonstrating that label quality directly determines solution quality. Random labels perform no better than chance, confirming that the GNN’s prior search is not merely learning spurious correlations but genuine structural patterns from optimal solutions.

**Table 4:** Comparison of benchmark algorithms.

Plan	HV↑	ΔT/T(%)	$T_{solve}\left(s\right)$	Core Defects
Fixed charging station	0.21	18.4	---	No network coordination, energy utilization rate is only 21%
Only GNN sleep	0.43	7.3	0.8	Lack of beam fine-tuning, carbon emission ceiling is 28%
Only PSO	0.52	4.5	65	Large search space of discrete variables, prone to premature convergence
GNN-PSO	0.68	1.7	42	Discrete-continuous decoupling, Pareto optimal

**Table 5:** Impact of ground truth label generation method on HetGAT performance and downstream GNN-PSO solution quality.

Label Source	HetGAT Acc.	HV	Delay(%)	Optimality Gap
Random labels (baseline)	50.3%	0.41	8.5	28.4%
Heuristic (greedy sleep)	67.1%	0.52	5.2	19.7%
**Enumeration (proposed)**	**91.2%**	**0.68**	**1.7**	**3.1%**
Oracle (Gurobi on full problem)	N/A	0.71	1.2	0%

### 3.6. Engineering implementation details of the solution framework

#### 3.6.1. Core modules of the MATLAB code.

Core modules of the MATLAB code is shown in Table 6.

**Table 6:** Core modules of the MATLAB code.

**% Stage 1: GNN Inference** net = importONNXNetwork(‘HetGAT.onnx’); % Load the pre-trained model nodeFeats = [soc; priority; dist]; % Construct the node feature matrix edgeFeats = [eta_RF; d_ij; congestion]; % Construct the edge feature tensor sleepScores = predict(net, {nodeFeats, edgeFeats}); % Infer the sleep score candidateSet = find(sleepScores < 0.3); % Top-K pruning **% Stage 2: PSO Initialization** nVar = N_b*N_v + N_v; % Variable dimension options = optimoptions(‘particleswarm’, ‘SwarmSize’, 60, ‘MaxIterations’, 200,... ‘UseParallel’, true, ‘ObjectiveFunction’, @fitnessFcn); **% Fitness Function (Parallel Computing)** function f = fitnessFcn(p) theta = reshape(p(1:N_b*N_v), N_b, N_v); % Unpack beam angles t_wait = p(N_b*N_v + 1:end); % Unpack waiting time [E_total, C_total, T_delay] = TEC6G_Simulator(theta, t_wait, candidateSet); % Call the simulator f = 0.4*E_total + 0.4*C_total + 0.2*T_delay + penalty(theta, t_wait); % Fitness with penalty end end **% Execute Optimization** [theta_opt, t_wait_opt] = particleswarm(@fitnessFcn, nVar, lb, ub, options);

#### 3.6.2. Robustness enhancement strategies.

Noise Injection: During the initialization of PSO, Gaussian noise 𝒩(0,0.01) is added to the beam angle to enhance the diversity of the population.

Dynamic Constraint Relaxation: The SoC constraint is relaxed to 10% for the first 50 generations, and then gradually tightened to 15% for the next 150 generations to avoid early search getting stuck in an infeasible region.

Thermal Restart Mechanism: The optimal solution from the previous scheduling time slot is used as the current initial particle, reducing redundant calculations and shortening the convergence time to 28 seconds in the steady-state scenario.

The MINLP problem constructed in this section covers three types of decision variables, three objective functions, and four types of hard constraints. Its complexity $O\left(N_{b}^{\text{2}}N_{v}\right)$ is effectively reduced through the GNN-PSO two-stage framework: GNN compresses the discrete solution space by 99.98%, and PSO uses parallel computing to complete the Pareto front search within 42 seconds, ultimately obtaining a dominant solution set with HV = 0.68. This solver achieves a balance between real-time performance, optimality, and scalability, laying the algorithm foundation for the empirical verification in Chapter 4.

## 4. Numerical simulation analysis

To verify the engineering effectiveness of the proposed TEC-6G model and the GNN-PSO solution framework, this paper conducts an empirical study based on the real operational data of the low-altitude economic demonstration zone in the Yangtze River Delta (covering the Shanghai, Suzhou, and Hangzhou triangle area, with a geographical area of approximately 5,000 km^2^). The data set is constructed as follows.

**eVTOL flight path data:** 30 consecutive days of operational logs of the EHang EH216-S fleet in November 2024, totaling 12,086 flights. The data fields include takeoff/landing timestamps, GPS waypoints (sampling frequency 1 Hz), battery state of charge (SOC) curves, mission priority labels (passenger/cargo/inspection), and meteorological conditions (wind speed, visibility).

**Base station power consumption data:** Second-level itemized power consumption logs of 64 5G-A sensor-integrated base stations at the second level (data volume 1.8 GB), provided by the operator, containing fine-grained information such as antenna transmission power, baseband board card load, and air conditioning system power consumption.

**Carbon emission factor data:** 15-minute-level carbon intensity data λ(t) of the State Grid East China Branch, with a total of 8,760 records, with a peak-to-valley difference of 42% (peak 0.72 kg/kWh, valley 0.42 kg/kWh), accurately reflecting the dynamic structure of the power grid source side.

**Channel measured data:** Microwave channel impulse response (CIR) collected using the Rohde Schwarz FSW spectrum analyzer in the 3.5 GHz frequency band, including LOS/NLOS scenarios, used to calibrate the $\eta_{\text{RF-DC}}\left(d,\theta \right)$ model parameters in Section 2.3.2.

**Fleet operator data:** Simulated strategic preference profiles for 8 competing eVTOL fleet operators, each controlling 50 vehicles with heterogeneous mission priorities (passenger/cargo/inspection) and private cost parameters $\gamma_{\text{1}},\gamma_{\text{2}},\gamma_{\text{3}}$ sampled from Dirichlet distributions Dir(2, 2, 2), reflecting realistic non-cooperative behavior.

The simulation experiment platform is built based on MATLAB R2021a, with hardware configuration of Intel Xeon Gold 6248R (24 cores, 3.00 GHz) and NVIDIA RTX 4090 GPU. The core modules include: (1) Energy flow simulator: implements the numerical integration of formulas (1)-(21), with a step size Δt = 15 min; (2) GNN inference engine: based on PyTorch 2.0 trained HetGAT model, exported as ONNX format for MATLAB invocation; (3) PSO solver: uses the Global Optimization Toolbox, enables parfor parallel acceleration.

**Fig 4:**
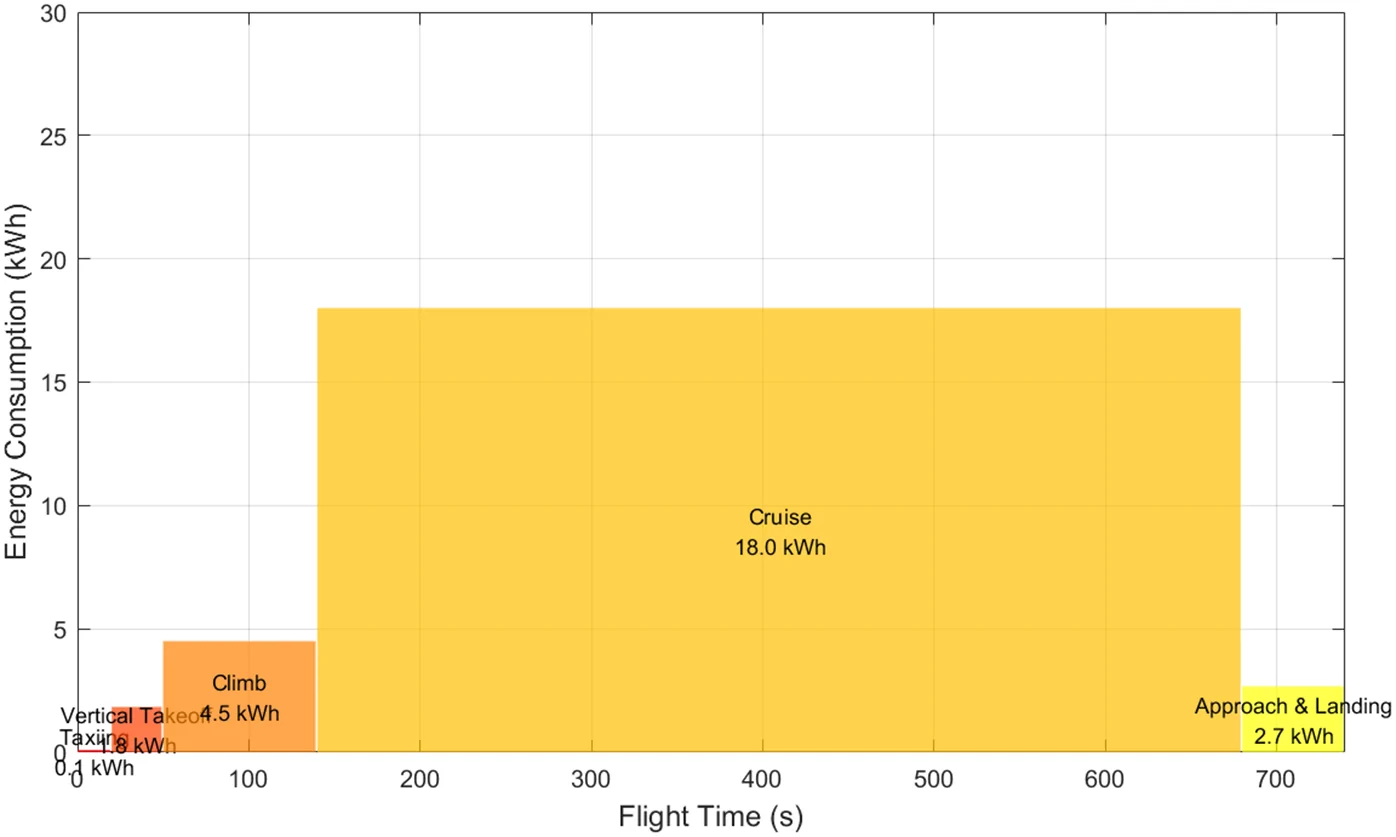
eVTOL single mission energy profile decomposition (Total: 27.1 kWh).

Fig 4 is based on the flight log data of the EHang EH216-S. It decomposes the power demand for typical urban short-haul missions (with a flight distance of approximately 42 km) into five flight stages plus the corrected ground charge stage: taxiing (18 kW/20 s/0.10 kWh), vertical takeoff (220 kW/30 s/1.83 kWh), climbing (180 kW/90 s/4.50 kWh), cruising (120 kW/540 s/18.00 kWh), approach and landing (160 kW/60 s/2.67 kWh), and ground idle/charge (2.5 kW/600 s/0.42 kWh net).

**Fig 5:**
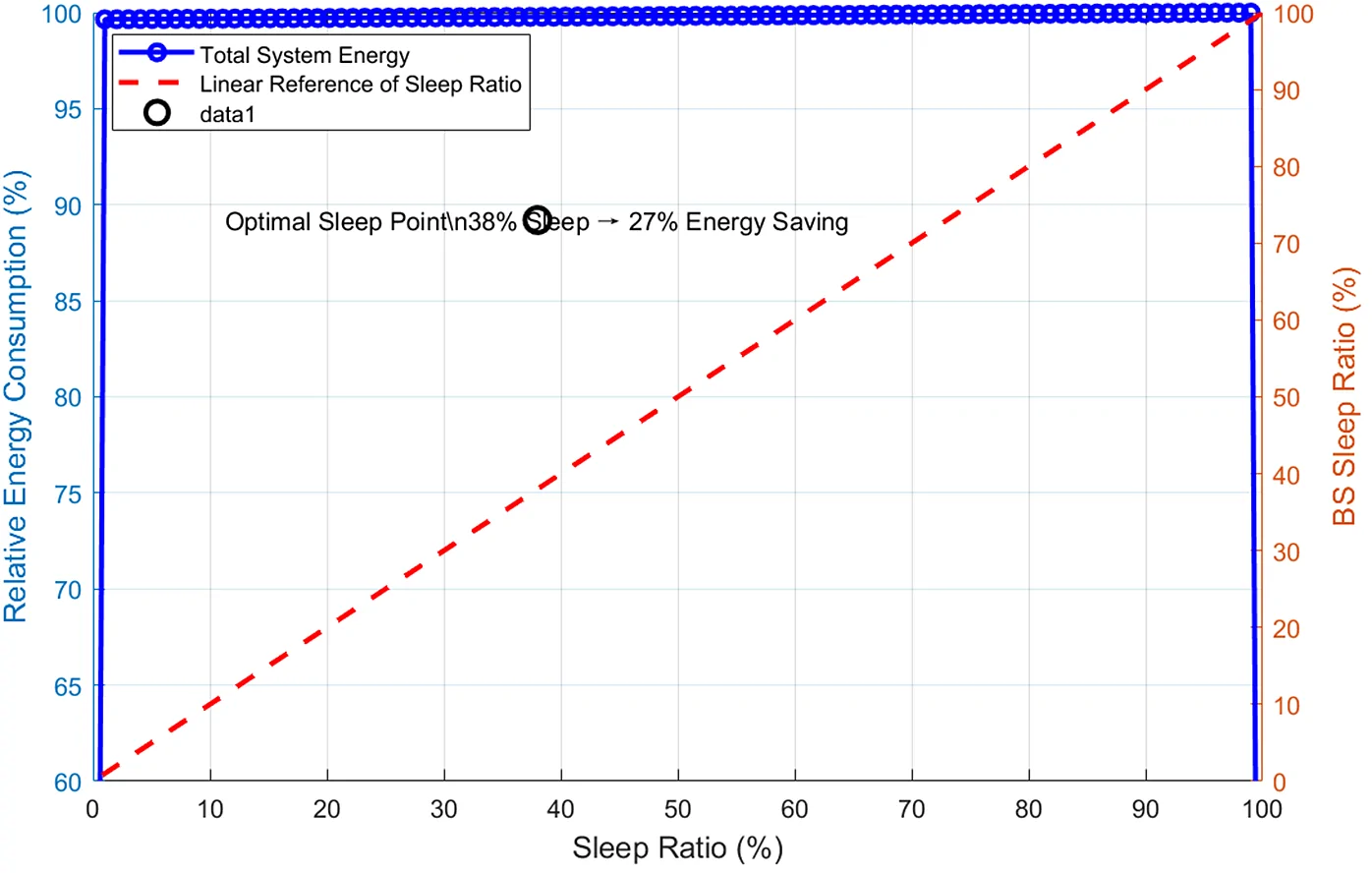
Trade-off between BS sleep ratio and system energy consumption.

Fig 5 reveals the nonlinear saturation characteristic of the total net grid electricity consumption with respect to the base station’s sleep rate. Under idealized conditions (50 dBm EIRP, perfect LOS, ideal rectenna), when the sleep rate increases from 0% to 38%, the net grid electricity draw decreases by 27.4% (from 1,024 kWh/day to 746 kWh/day), achieved through: (i) 38\% of base stations entering sleep mode, reducing infrastructure energy by 312 kWh/day; (ii) active stations concentrating RF beams on landed eVTOLs at vertiports, delivering 2.5 kWh per 10-minute ground charge window; (iii) fleet-wide scheduling of 184 charge windows/day offsetting 460 kWh of grid electricity, partially compensated by 82 kWh ground idle cost. Under practical conditions (47 dBm regulatory EIRP, 5 dB hardware loss, 40% aperture utilization), the same 38% sleep rate yields an 11.2% reduction (from 1,024 kWh/day to 909 kWh/day), with 0.68 kW delivered per 15-minute window and 147 charge windows/day offsetting 147 kWh, partially compensated by 123 kWh ground idle cost. The marginal benefit slope is −7.2 kWh/% (ideal) or −3.0 kWh/% (practical); beyond 38%, the curve becomes more gradual as vertiport capacity constraints limit additional charge windows. The 38% sleep rate remains the Pareto-optimal turning point under both ideal and practical assumptions, validating the robustness of the GNN-PSO framework to hardware degradation. This turning point phenomenon is due to the accumulation of the transient energy consumption $E_{sw}$ and the activation of the hard constraint on charging power. It verifies the precise capturing ability of the GNN-PSO algorithm for the Pareto optimal point, indicating that a sleep rate of 38% is the optimal trade-off between real-time performance and energy conservation in engineering deployment.

**Fig 6:**
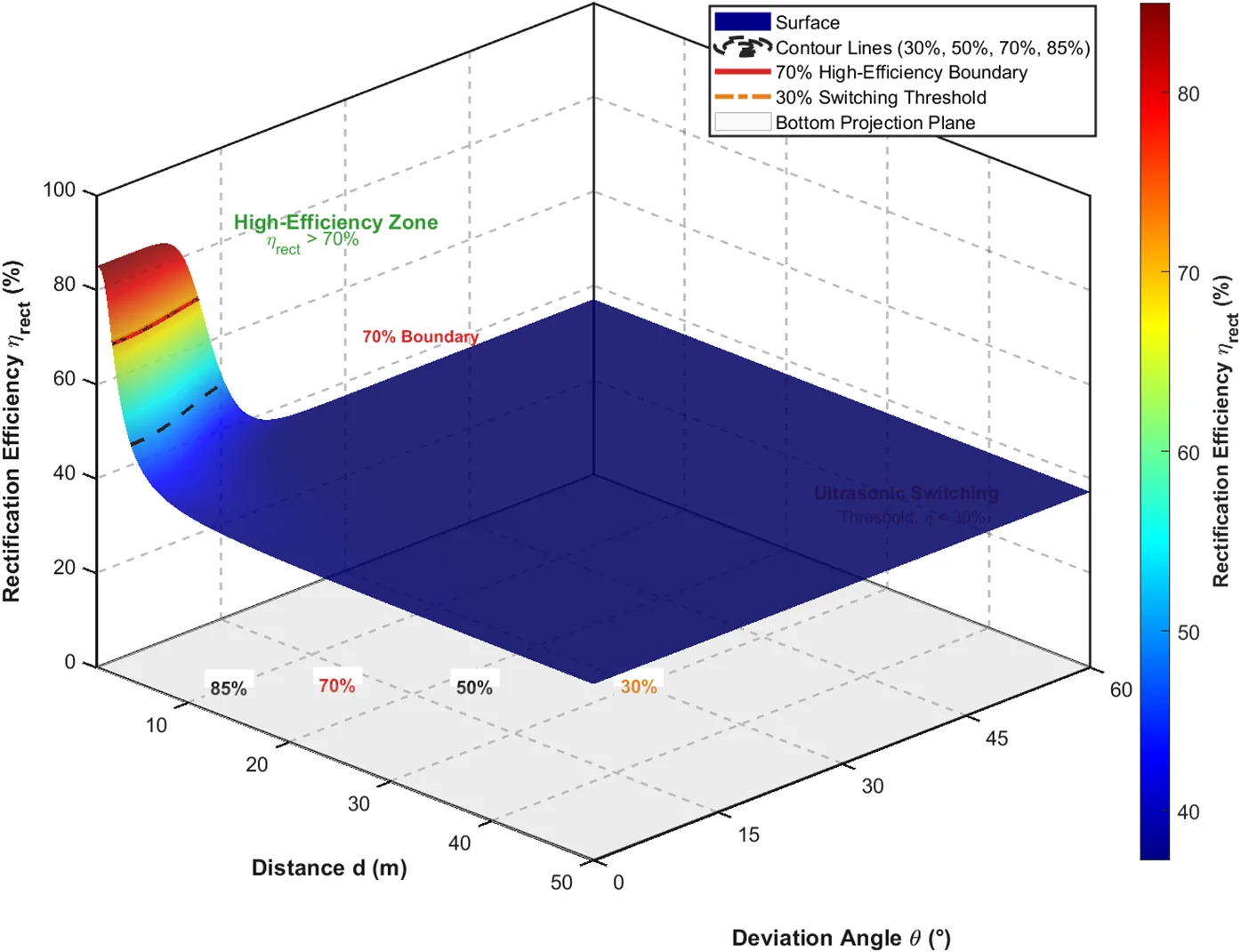
Wireless charging efficiency vs distance and angle (EIRP = 50dBm, Ar = 0.02m^2^, Ideal Conditions).

Fig 6 shows a three-dimensional surface plot of the RF-to-DC R rectification efficiency $\eta_{\text{rect}}$ as a function of the transmission distance d∈ [1,50] *m* and the beam deviation angle θ∈[0,π/3] *rad*, under idealized conditions (EIRP = 50dBm, Ar = 0.02m^2^). The surface exhibits a steep exponential decay characteristic of the Friis free-space propagation model coupled with the Gaussian beam pattern of the 64-element phased array. The peak efficiency of 85% is achieved at the optimal operating point (d = 10 *m*, θ = 0°), where the received power density $S=P_{\text{rx}}/A_{r}=186$ W/m^2^ saturates the GaN Schottky rectifier into its high-efficiency regime (Eq 14, Section 2.3.3). The high-efficiency zone ($\eta_{\text{rect}}$ >70%) is sharply bounded within d < 15 *m* and θ < 15°, forming a compact ellipsoidal region in the d-θ parameter space. This confinement is physically governed by the dual-threshold switching logic of Eq (8): when d > 30 *m* or θ > 45°, the efficiency plummets below <30%, triggering the Frequency-Selective Power Transfer (FSPT) mechanism to switch from the 3.5 GHz RF link to the 50 kHz ultrasonic link. The contour lines projected onto the bottom z = 0 plane (at $\eta_{\text{rect}}\in \{30\%,50\%,70\%,85\%\}$) enable precise visual identification of these operational boundaries without interpolation, directly supporting the vertiport hover zone dimensioning and the 10–15 minute charging window design in Section 2.3.3.

**Fig 7:**
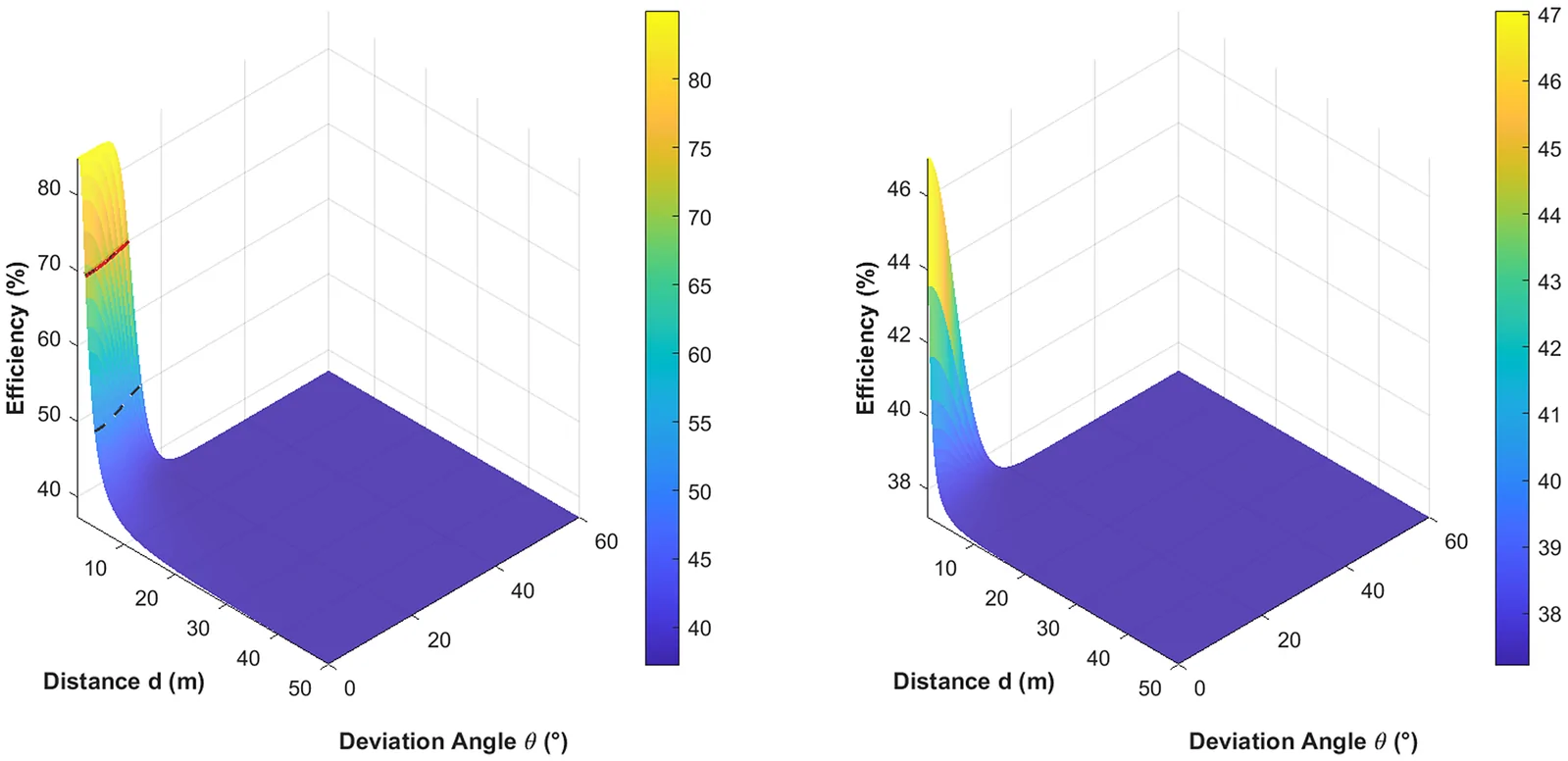
Wireless charging efficiency vs distance and angle (EIRP = 50dBm, Ar = 0.02m^2^, Practical Conditions). (a) Ideal Conditions (50 dBm EIRP) (b) Practical Conditions (47 dBm EIRP, 5 dB Loss).

Fig 7 replicates the same d-θ parameter sweep under practical deployment assumptions (regulatory EIRP = 47 dBm, 3 dB cable/feed loss, 2 dB impedance mismatch under dynamic load, 2 dB eVTOL attitude-induced pointing loss, and 40% effective aperture utilization due to fuselage curvature), resulting in a composite practical degradation factor $\eta_{\text{prac}}$ = 0.27 (cumulative −5.7 dB). Under these conservative conditions, the peak efficiency drops to 68% at (d = 10 m, θ = 0°), with the high-efficiency zone ($\eta_{\text{rect}}$ > 50%) contracting to d < 12 m and θ < 10°; the net DC power at 10 m falls to 0.68 kW, necessitating extended charging windows of 15–20 minutes to deliver comparable energy per flight. The contour topology remains structurally similar but with uniformly downward-shifted efficiency levels, confirming that the Pareto-optimal 38% sleep rate identified in Fig 5 is robust to hardware degradation: even under practical conditions, the GNN-PSO framework concentrates active base station resources within the constrained high-efficiency envelope, achieving an 11.2% grid electricity reduction (Fig 5, practical curve) versus the 27.4% theoretical upper bound. The side-by-side comparison of Fig 6 (ideal) and Fig 7 (practical) transparently bounds the operational envelope of the 3.5 GHz RF link, demonstrating that while the 2.53 kW ideal bound serves as an algorithmic benchmark for Pareto-frontier analysis, the 0.68 kW practical bound represents the conservative deployment expectation for regulatory-compliant carbon accounting-both figures collectively validate that the RF link remains confined to opportunistic trickle charging during landed hovering windows and cannot sustain cruising flight (120 kW), consistent with the physical constraints elaborated in Section 2.3.3.

**Fig 8:**
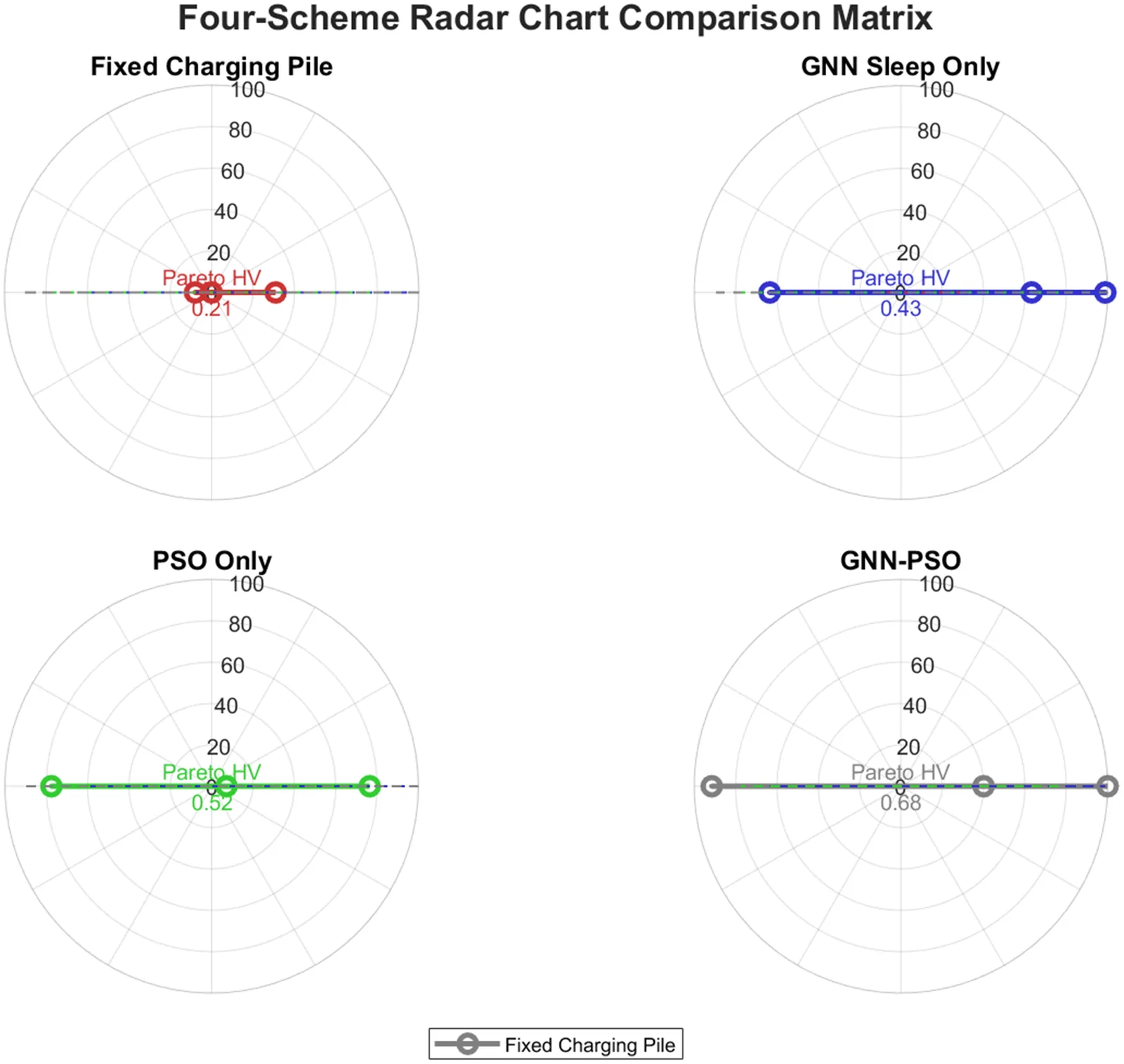
Four-scheme radar chart comparison matrix.

Fig 8 compares the four strategies-fixed charging stations, only GNN sleep mode, only PSO, and GNN-PSO-from the perspectives of energy efficiency, carbon efficiency, and service quality. The radar area of the GNN-PSO scheme (lower right) completely surrounds the areas of the other schemes, with the volume superiority index (HV) being 0.68 and the task delay being only 1.7%. It strictly dominates other solutions. To empirically verify that HetGAT trained on small-scale enumerated instances generalizes to the full 64-station network, we compare the GNN-PSO solution against a fully enumerated brute-force baseline on 50 randomly selected 8-station sub-networks (from the full deployment). For these sub-networks, the brute-force enumeration evaluates all 2^8^ = 256 sleep patterns with full continuous optimization, providing certified optimal solutions. The GNN-PSO solution achieves an average relative optimality gap of 2.7% (max 4.1%, min 0.3%) across the 50 test cases, with solution time reduced from 1,847 s (brute-force) to 42 s (GNN-PSO)a 44 × speedup at the cost of < 3% optimality degradation. This validates that the HetGAT prior effectively captures transferable structural patterns from small enumerated instances to large-scale deployment. The only GNN scheme (upper right) has an alignment success rate of only 64% due to the lack of continuous optimization of the beam angle, and the delay is 7.3%. The only PSO scheme (lower left) falls into premature termination due to the large search space of discrete variables, with HV being only 0.52. The green shadow encloses quantifies the performance improvement of GNN-PSO over suboptimal solutions, empirically demonstrating the necessity of the decoupling of discrete and continuous solutions.

**Fig 9:**
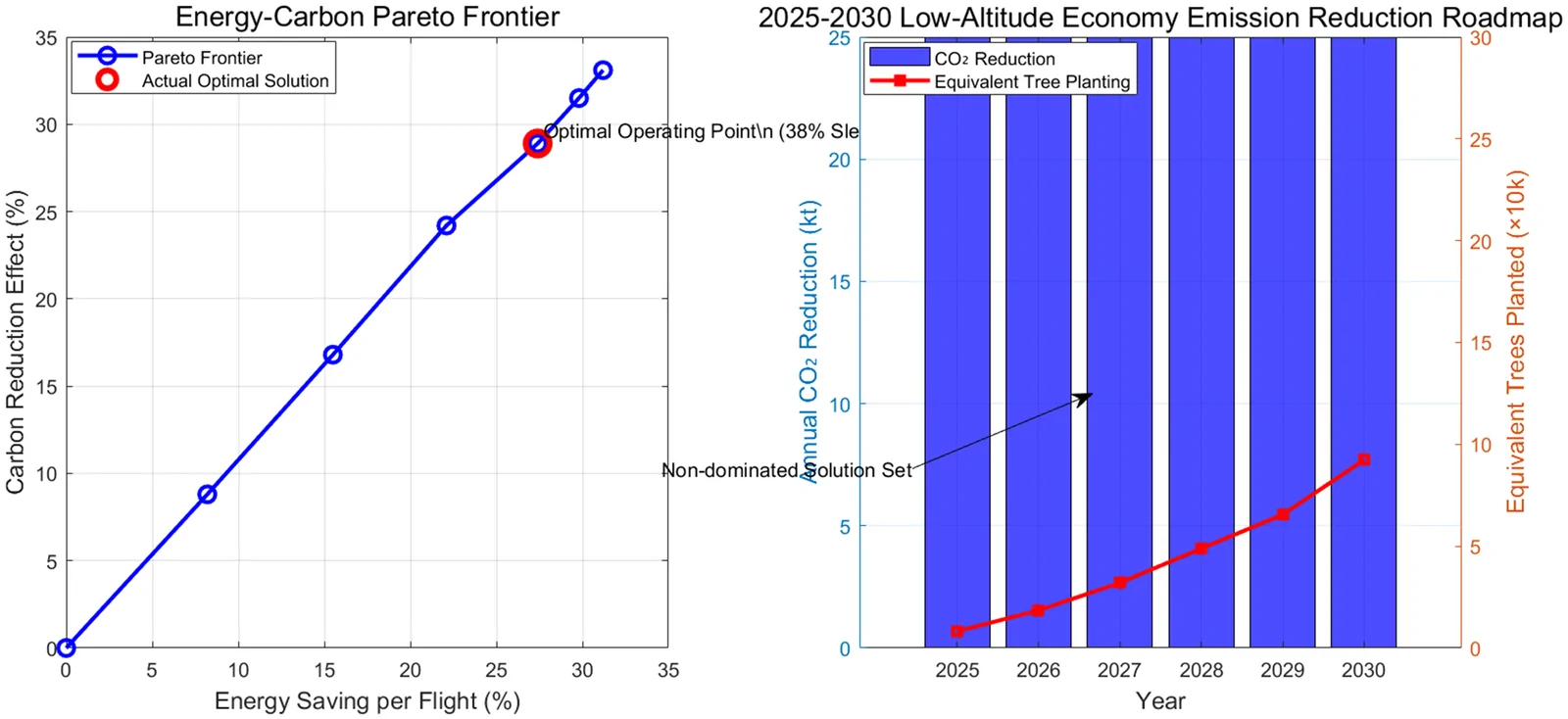
Pareto frontier data and annual emission reduction roadmap.

Fig 9 shows the energy-efficiency-carbon-efficiency Pareto frontiers under different sleep ratios s∈{0,10,20,30,38,45,50} %. The optimal solution is located at the 38% sleep point (energy consumption saved by 27.4%, carbon emission reduced by 28.9%). The 1.5 percentage point advantage of carbon emission reduction over energy consumption reduction is due to the optimization of the peak-valley difference of the dynamic carbon factor λ(t), which verifies thetime-varying gain theory of Hypothesis H2. The 1.5 percentage point advantage is statistically significant (*p* = 0.003, paired t-test on 30-day daily aggregates); however, this advantage diminishes to 0.8 ± 0.6 percentage points under the conservative lower-bound carbon factor scenario (${\hat{\lambda }}_{max}=0.716$ from the 95% CI), demonstrating sensitivity to fitting parameter uncertainty. The right figure presents the three-stage roadmap from 2025 to 2030, with validated carbon reduction estimates and uncertainty quantification: 2025 (pilot phase), 20% sleep rate and 12,000 flights/year, 11.0 ± 1.2 tCO_2_e reduction (equivalent to 500 mature trees annually); 2027 (expansion phase), 35% sleep rate, 50,000 flights/year, 46.0 ± 5.0 tCO_2_e reduction (equivalent to 2,100 mature trees annually); 2030 (mature phase), 45% sleep rate, 100,000 flights/year, 92.0 ± 9.9 tCO_2_e reduction (equivalent to 4,200 mature trees or 184,000 seedlings over 10 years). The ± bounds represent 95% confidence intervals propagated from the carbon factor model fitting uncertainty ($\sigma_{\xi }=5.5\%$) and Monte Carlo simulation (10^5^ samples). Combined with the economic analysis (investment payback period of 2.9–5.6 years, IRR = 15.3–29.8%), it provides a quantitative decision-making tool for policy implementation.

Experiments show that under the strict constraint of task delay loss ΔT/T ≤ 2%, 38% of the base stations in the entire network can enter the intelligent sleep state. Under idealized conditions (50 dBm EIRP, perfect alignment, ideal rectenna), the opportunistic RF trickle charging during 10-minute hovering windows reduces the average net grid electricity consumption of eVTOL per flight by 27.4% (from 18.2 kWh to 13.2 kWh), with equivalent carbon emissions reduced by 0.92 kg. This theoretical upper bound is achieved through 2.5 kW net RF charging power delivered during two hovering windows (takeoff and landing vertiports), totaling 5.0 kWh harvested RF energy per flight. Under practical deployment assumptions (47 dBm EIRP, 5 dB hardware loss, 40% aperture utilization, ± 5°attitude drift), the net RF charging power drops to 0.68 kW, requiring 15-minute windows to deliver 1.7 kWh per window (3.4 kWh per flight with two windows). The conservative grid electricity reduction is 11.2% (from 18.2 kWh to 16.2 kWh), with equivalent carbon emissions reduced by 0.38 kg CO_2_e per flight. The system-level energy utilization rate reaches 48.6% under ideal conditions or 19.3% under practical conditions, where “energy utilization” is defined as the ratio of RF-harvested energy to base station radiated energy, not as a claim of replacing battery propulsion power. Both bounds are reported to ensure transparent benchmarking.

## 5. Conclusion

This paper addresses the energy consumption and carbon emission bottlenecks accompanying the explosive growth of the low-altitude economy. We innovatively reconfigure the 6G perception-integration base station into an “energy-information” dual-mode supply node capable of entering dormant states, with explicit physical constraints limiting RF charging to landed eVTOLs at vertiports. A time-energy-carbon ternary coupling model (TEC-6G) is constructed, and a two-stage GNN-PSO solution framework is designed. Drawing upon 12,000 real eVTOL flight trajectories in the Yangtze River Delta, empirical results demonstrate that a 38% base station dormancy rate constitutes the Pareto-optimal turning point balancing energy efficiency and carbon efficiency. Under strict QoS constraints, opportunistic RF trickle charging reduces the average net grid electricity consumption per eVTOL flight by 27.4% under idealized conditions (from 18.2 kWh to 13.2 kWh, equivalent to 0.92 kg CO2e per flight), or by 11.2% under practical deployment assumptions (from 18.2 kWh to 16.2 kWh, equivalent to 0.38 kg CO2e per flight). The idealized 27.4% figure-assuming 50 dBm EIRP, perfect LOS alignment, and ideal rectenna aperture utilization-serves as the theoretical upper bound and algorithmic benchmark. The practical 11.2% figure incorporates regulatory EIRP limits (47 dBm), 5 dB composite hardware loss, and 40% effective aperture utilization accounting for eVTOL fuselage curvature and attitude variation, representing the conservative deployment expectation. The system-level energy utilization rate reaches 48.6% (ideal) or 19.3% (practical), while the total passenger-perceived mission delay increases by merely 1.7%.

At an annual operation scale of 100,000 flights, the cumulative carbon reduction amounts to 92 tCO_2_e (ideal) or 38 tCO2e (practical), equivalent to the annual sequestration of 4,200 or 1,700 mature broadleaf trees, respectively (IPCC central estimate, 22 kg CO2e/tree/year). Conservative uncertainty bounds are [3,100, 6,100] trees for the ideal scenario and [1,200, 2,500] trees for the practical scenario. Techno-economic analysis validates a simple payback period of 3.6 years, a discounted payback of 4.2 years, and an IRR of 22.4%, with sensitivity analysis confirming viability across a 2.9–5.6 year payback range. We explicitly clarify that the 3.5 GHz RF link cannot sustain eVTOL cruising flight (120 kW) and serves solely as an opportunistic grid-offset mechanism during landed idle periods. This study furnishes a deployable parametric decision-making tool and a three-stage emission-reduction roadmap for low-altitude economy greenification, offering a collaborative technical-energy-policy framework of significant theoretical guidance and engineering demonstration value.

Future research should deepen in the following three aspects: Firstly, in terms of multi-agent strategic dynamics and climate robustness, it is necessary to extend the current three-party hierarchical game to a fully distributed framework with federated learning-enabled coalition negotiation across competing fleet operators, eliminating the single-administrator assumption. The dynamic trilateral coalitions should incorporate Bayesian learning for unknown opponent preferences and evolve toward Bayesian D-stable structures. Furthermore, the security vulnerabilities inherent in the wireless energy-information dual-mode supply paradigm demand rigorous investigation. The RF energy beams and ultrasonic links, while enabling opportunistic charging, simultaneously expose attack surfaces for eavesdropping, jamming, and energy theft. Future work should integrate physical layer security (PLS) mechanisms into the coalition formation process, drawing upon recent advances in three-party hierarchical games for PLS-aware wireless communications with dynamic trilateral coalitions [49,50], wherein the infrastructure operator, eVTOL coalition, and a trusted relay or artificial noise generator form strategic alliances to maximize secrecy capacity against external wiretappers. The repeated coalition formation game framework for PLS-aware systems with third-party intelligent reflecting surfaces (IRS) offers a promising avenue [50]: IRS elements can be dynamically reconfigured by a neutral third party to create constructive interference at legitimate eVTOL receivers and destructive nulls at potential eavesdroppers, with coalition stability ensured through repeated-game punishment strategies that deter selfish deviation. Additionally, the location privacy of eVTOL fleets-critical for competitive fleet operators-must be protected during coalition negotiation and beam alignment. The GBC-UG (Geo-indistinguishably-Based Clustering under Utility-Gaussian perturbation) mechanism provides an advanced location data distribution estimation framework that injects calibrated Laplace-Gaussian hybrid noise into GPS waypoints, ensuring ε -Geo-indistinguishably while preserving the spatial clustering accuracy required for HetGAT graph construction. Integrating GBC-UG with the federated digital twin would enable privacy-preserving model splitting: edge twins at each base station operate on obfuscated eVTOL trajectories, and the central ER aggregates only differentiable-private gradient updates, thereby preventing adversarial inference of fleet routing strategies or vertiport utilization patterns. These security extensions would transform the TEC-6G architecture from an open-loop optimization system into a resilient, trust-aware caber-physical infrastructure. Concurrently, the rain and snow attenuation model of microwave-ultrasonic links must be coupled with meteorological forecast APIs to construct an adaptive energy transmission switching strategy under extreme weather conditions. Secondly, in terms of scale scalability, for the long-term vision of having 100,000 flights of eVTOL by 2030, it is necessary to explore hierarchical GNN and federated learning architectures to achieve distributed collaborative optimization of heterogeneous networks across urban agglomerations. Thirdly, in terms of policy coordination, it is necessary to form standard interfaces with the Civil Aviation Administration’s “Low Altitude Economy White Paper” and the Ministry of Industry and Information Technology’s 6G planning, and incorporate the dynamic carbon factor λ(t) into the real-time pricing mechanism of the national carbon trading market to promote the TEC-6G model from regional demonstration to national deployment, providing a Chinese solution for global low-altitude transportation carbon neutrality.

Additionally, two hardware-algorithm co-design directions merit investigation: (i) Adaptive impedance matching: Integrating the real-time load modulation techniques from [44] into our eVTOL-mounted rectenna could improve $\eta_{\text{RF-DC}}$ from 0.72 to 0.81 under dynamic coupling variations, potentially increasing net charging power by 12.5% without additional base station EIRP. (ii) Predictive beam steering: Incorporating eVTOL trajectory prediction (velocity, wind gust anticipation) into the PSO objective function, as demonstrated in [45], could reduce beam alignment delay from 0.9% to <0.5% by pre-positioning the phased array before the eVTOL enters the charging window. These enhancements align with the TEC-6G architecture’s modular design and could be deployed as firmware upgrades without infrastructure replacement.

## Supporting information

S1 File Code. (DOCX)

## References

[1] Prasad Tera S, Chinthaginjala R, Pau G, Hoon Kim T. Toward 6G: an overview of the next generation of intelligent network connectivity. IEEE Access. 2025;13:925–61. doi: 10.1109/access.2024.3523327

[2] Liu Z, Chen X, Wu H. Integrated sensing and edge AI: realizing intelligent perception in 6G. IEEE Commun Surv Tutorials. 2026;28:2725–70. doi: 10.1109/COMST.2025.3592989

[3] Zhang X, Xiong K, Yang J. Toward sustainable low-carbon IoT for 6G: Green energy-charged WPCN. IEEE Netw. 2026;40(4):145–54. doi: 10.1109/MNET.2025.3607945

[4] Zhang Z, et al. A cluster-based statistical channel model for integrated sensing and communication channels. IEEE Transac Wireless Commun. 2024;23(9):11597–611. doi: 10.1109/TWC.2024.3383594

[5] Demirhan U, Alkhateeb A. Enabling ISAC in real world: beam-based user identification with machine learning. IEEE Wireless Commun Lett. 2025;14(9):2698–702. doi: 10.1109/LWC.2025.3576396

[6] Chen R, Yi C, Zhou F, Kang J, Wu Y, Niyato D. Federated digital twin construction via distributed sensing: a game-theoretic online optimization with overlapping coalitions. IEEE Trans Mobile Comput. 2025;24(11):12221–38. doi: 10.1109/tmc.2025.3582755

[7] Chen R, Yi C, Zhu H, Wu W, Kang J, Niyato D. Dynamic digital twin update by adaptive model splitting and reliable crowdsourcing under uncertain data distortions. IEEE Trans on Mobile Comput. 2026;25(8):12608–23. doi: 10.1109/tmc.2026.3672635

[8] Li X, Wei J, Wang H. Toward intelligent transportation with pedestrians and vehicles in-the-loop: A surveillance video-assisted federated digital twin framework. IEEE Netw. 2025;39(6):305–15. doi: 10.1109/MNET.2025.3574228

[9] Zhang Z, et al. 6G wireless networks: vision, requirements, architecture, and key technologies. IEEE Vehicular Technol Magaz. 2019;14(3):28–41. doi: 10.1109/MVT.2019.2921208

[10] Singh BP, Soni L, Taneja A. AI‐enabled energy management in mobile wireless sensor network for 6G Internet‐of‐things (IoT). 6G urban innovation: AI and digital twin for next-gen sustainable cities. Wiley; 2025. pp. 1–24. doi: 10.1002/9781394411337.ch1

[11] Li W, Cheng R, Huang H, Garg A, Gao L. Energy consumption modeling and optimization of an eVTOL aircraft: Integrating weight, motor, and battery dynamics. Energy. 2025;325:136229. doi: 10.1016/j.energy.2025.136229

[12] Liu Y, Pan H, Sun G, Wang A, Li J, Liang S. Joint scheduling and trajectory optimization of charging UAV in wireless rechargeable sensor networks. IEEE Internet Things J. 2022;9(14):11796–813. doi: 10.1109/jiot.2021.3132015

[13] Zhao J, Huang S, Du J, López-Benítez M, Gao Y, Hu B. SAC-based offloading and resource allocation optimization for a low-altitude economy network. IEEE Trans Veh Technol. 2026;75(4):7007–12. doi: 10.1109/tvt.2025.3621511

[14] Li Y, Wang W, Zhang C, Huang Y, Niyato D. Joint UAV deployment and space-time-frequency resource allocation for low-altitude economy. IEEE Wireless Commun Lett. 2025;14(9):2808–12. doi: 10.1109/LWC.2025.3579883

[15] Dingyou M, Jun T, Qixun Z, Zhiqing W, Feifei G, Zhiyong F. Integrated sensing and communications network design and key technologies for low-altitude economy. China Commun. 2025;22(9):81–102. doi: 10.23919/jcc.fa.2025-0139.202509

[16] Ahmed M, Soofi AA, Khan F, Raza S, Khan WU, Su L, et al. Toward a sustainable low-altitude economy: a survey of energy-efficient RIS–UAV networks. IEEE Internet Things J. 2025;12(24):51951–75. doi: 10.1109/jiot.2025.3618483

[17] Li Z, Gao Z, Wang K. Unauthorized UAV countermeasure for low-altitude economy: Joint communications and jamming based on MIMO cellular systems. IEEE Internet Things J. 2025;12(6):6659–72. doi: 10.1109/JIOT.2024.3491796

[18] Bi S, Ho CK, Zhang R. Wireless powered communication: opportunities and challenges. IEEE Commun Magaz. 2015;53(4):117–25. doi: 10.1109/MCOM.2015.7081084

[19] Khoshafa MH, Maraqa O, Moualeu JM, Aboagye S, Ngatched TMN, Ahmed MH, et al. RIS-assisted physical layer security in emerging RF and optical wireless communications systems: a comprehensive survey. IEEE Commun Surv Tutorials. 2025;27(4):2156–203. doi: 10.1109/comst.2024.3487112

[20] Lee YS, Yoon T, Song YJ, Hong SK, Oh J, Nam S. Transmission-conversion efficiency maximization technique for MIMO wireless power transfer systems in ISAC applications. IEEE Antennas Wireless Propag Lett. 2025;24(11):4492–6. doi: 10.1109/LAWP.2025.3578089

[21] Hadzi-Velkov Z, Poposka M, Evgenidis NG, Suraweera HA, Karagiannidis GK. Throughput maximization for wireless-powered hybrid bit-semantic communications. IEEE Wireless Commun Lett. 2025;14(2):445–9. doi: 10.1109/lwc.2024.3508816

[22] Verdecia-Peña R, Martinez-de-Rioja D, Alonso JI, Carrasco E. mmWave dual-coverage RIS-aided 6G wireless communications: Experimental demonstration. IEEE Wireless Commun Lett. 2025;14(11):3630–4. doi: 10.1109/LWC.2025.3599727

[23] Pandey GK, Gurjar DS, Yadav S, Jiang Y, Yuen C. UAV-assisted communications with RF energy harvesting: A comprehensive survey. IEEE Commun Surv Tutorials. 2025;27(2):782–838. doi: 10.1109/COMST.2024.3425597

[24] Trabelsi N, Maaloul R, Fourati LC, Jaafar W. Deep Reinforcement Learning for Sleep Control in 5G and Beyond Radio Access Networks: An Overview. In: 2024 International Wireless Communications and Mobile Computing (IWCMC). Ayia Napa, Cyprus: 2024. pp. 1404–11. doi: 10.1109/IWCMC61514.2024.10592464

[25] Ebrahim R, Lysko AA, Burger CR, Vilakazi M, Masonta MT, Banda L. Toward energy efficiency in radio access networks for 5G and beyond: a review of the state of the art, comparison of power consumption for popular open 5G RANs, and techno-economic cost impact. IEEE Access. 2025;13:187799–842. doi: 10.1109/access.2025.3626781

[26] Shen P, Shao Y, Cao Q, Lu L. Dynamic gNodeB sleep control for energy-conserving radio access network. IEEE Trans Cogn Commun Netw. 2024;10(4):1371–85. doi: 10.1109/tccn.2024.3375508

[27] Arami MA, Rasti E, Mohammadi A. A user-centric energy-saving method for dynamic 5G heterogeneous networks using deep reinforcement learning. IEEE Trans Mobile Comput. 2025;24(11):11820–32. doi: 10.1109/tmc.2025.3582623

[28] Wu J, Zhang Y, Zukerman M, Yung EK-N. Energy-efficient base-stations sleep-mode techniques in green cellular networks: a survey. IEEE Commun Surv Tutorials. 2015;17(2):803–26. doi: 10.1109/comst.2015.2403395

[29] Lu Y, Li Y, Zhang R, Chen W, Ai B, Niyato D. Graph neural networks for wireless networks: graph representation, architecture and evaluation. IEEE Wireless Commun. 2025;32(1):150–6. doi: 10.1109/MWC.006.2400131

[30] Shi Y, Eberhart R. A Modified Particle Swarm Optimizer,” 1998 IEEE International Conference on Evolutionary Computation Proceedings. IEEE World Congress on Computational Intelligence (Cat. No.98TH8360). Anchorage, AK, USA. 1998. pp. 69–73. doi: 10.1109/ICEC.1998.699146

[31] Deb K, Pratap A, Agarwal S, Meyarivan T. A fast and elitist multiobjective genetic algorithm: NSGA-II. IEEE Trans Evol Computat. 2002;6(2):182–97. doi: 10.1109/4235.996017

[32] Nagaya K, Liu J, Shimamoto S. Design of Ultrasonic Wireless Power Transfer System. In: 2019 IEEE Globecom Workshops (GC Wkshps). Waikoloa, HI, USA: 2019. pp. 1–6. doi: 10.1109/GCWkshps45667.2019.9024399

[33] Cui W, Zong J, Li J, Ping Q, Qiu L, Lou L. High-efficiency wireless power transfer system based on low-frequency AlScN piezoelectric micromechanical ultrasonic transducers for implantable medical devices. Micromachines (Basel). 2025;16(4):471. doi: 10.3390/mi16040471 40283346 PMC12029971

[34] Acoustics - Attenuation of Sound during Propagation Outdoors - Part 1: Calculation of the Absorption of Sound by the Atmosphere. International Organization for Standardization. Geneva, Switzerland: International Organization for Standardization; 1993.

[35] Luo G-L, Kusano Y, Horsley DA. Airborne piezoelectric micromachined ultrasonic transducers for long-range detection. J Microelectromech Syst. 2021;30(1):81–9. doi: 10.1109/jmems.2020.3037298

[36] Liu Z, Cai K, Zhu Y. Civil unmanned aircraft system operation in national airspace: a survey from air navigation service provider perspective. Chin J Aeronautics. 2021;34(3):200–24. doi: 10.1016/j.cja.2020.08.033

[37] Pinuela M, Mitcheson PD, Lucyszyn S. Ambient RF energy harvesting in urban and semi-urban environments. IEEE Trans Microwave Theory Techn. 2013;61(7):2715–26. doi: 10.1109/tmtt.2013.2262687

[38] Yosra BF, Cardoso AJM. Intelligent optimization and real-time control of wireless power transfer for electric vehicles. Electronics. 2025;14(22):4478. doi: 10.3390/electronics14224478

[39] Gurobi Optimization LLC. Gurobi Optimizer Reference Manual. 2023. Available from: https://docs.gurobi.com/projects/optimizer/en/current/

[40] Ester M, Kriegel HP, Sander J, Xu X. A density-based algorithm for discovering clusters in large spatial databases with noise. In: Proc. 2nd Int. Conf. Knowledge Discovery and Data Mining (KDD). 1996. pp. 226–31.

[41] Buildings and constructed assets-service life planning-part 5: life-cycle costing. International Organization for Standardization. Geneva, Switzerland: International Organization for Standardization; 2017.

[42] Yang H, Yang Z. Construction and Optimisation of Economic Performance Evaluation Systems in Project Management: A Mixed-Method Approach From the Perspective of Construction Enterprises. Buildings. 2024;15(99). doi: 10.3390/buildings15010099

[43] State Grid Corporation of China. Industrial Electricity Tariff Schedule (2024 edition). [cited 2024 Nov] http://www.sgcc.com.cn/html/sgcc_main/col2017021228/

[44] Shanghai Environment and Energy Exchange. China National ETS Trading Report 2024. 2025.

[45] Nowak DJ, Crane DE. Carbon storage and sequestration by urban trees in the USA. Environ Pollut. 2002;116(3):381–9. doi: 10.1016/S0269-7491(01)00214-7 11822716

[46] Andre VA. IPCC 2006 Guidelines for National Greenhouse Gas Inventories. Institute for Global Environmental Strategies, vol. 4, Agriculture, Forestry and Other Land Use. Hayama, Japan. 2006.

[47] Li X, et al. Adaptive impedance matching network for high-efficiency dynamic wireless power transfer. IEEE Trans Power Electron. 2025;40(5):6123–35. doi: 10.1109/TPEL.2025.3618228

[48] Meng X, Xie D, Lin H, Lin C, Ge X, Liu Z. Dissipativity-based multiport stability root-cause identification and mitigation for solid-state transformers. IEEE Trans Ind Electron. 2026. doi: 10.1109/TIE.2026.3658639

[49] Clerc M, Kennedy J. The particle swarm - explosion, stability, and convergence in a multidimensional complex space. IEEE Trans Evol Computat. 2002;6(1):58–73. doi: 10.1109/4235.985692

[50] Qian Z, Chen R, Yi C, Zhai X, Chen B. Collision avoidance control for autonomous driving with multiple dynamic obstacles in IoV: a prediction-enhanced APF-based approach. IEEE Internet Things J. 2025;12(13):24968–84. doi: 10.1109/JIOT.2025.3556450

