# Supplementary material for: The 6G integrated perception and networking technology enables the low-altitude economy to achieve carbon neutrality: Dynamic sleep strategy for eVTOL wireless charging based on time-energy-carbon coupling
Source: PLoS One. 2026 Sep 16;21(9):e0347398. doi: 10.1371/journal.pone.0347398 (PMC13581032; doi:10.1371/journal.pone.0347398)
Supplement: Supplementary file 1 [file pone.0347398.s001.docx]

% =========================================================================

% Figure 2

% =========================================================================

clear; clc; close all;

%% UPT Parameters (from Section 2.3.3)

f_US = 50e3; % Ultrasonic frequency (Hz)

Ptx_US = 500; % Ultrasonic transmit power (W)

eta_trans = 0.85; % Electro-acoustic conversion efficiency

eta_rect = 0.65; % Piezoelectric receiver rectification efficiency

Ar = 0.02; % Effective receiving aperture (m^2)

% Standard atmospheric condition

T_std = 20; % Standard temperature (°C)

RH_std = 50; % Standard relative humidity (%)

alpha0 = 0.9; % Standard attenuation coefficient (dB/m)

% ISO 9613-1 approximation model

% alpha = alpha0 * (1 + 0.008*(T - T_std)) * (1 + 0.005*(RH - RH_std))

%% Define atmospheric conditions for subplots

conditions = {

'Standard (20°C, 50%RH)', [20], [50];

'Low Temperature (5°C, 30%RH)', [5], [30];

'High Temperature (35°C, 80%RH)', [35], [80];

'Full Envelope Comparison', [5,20,35], [30,50,65,80]

};

% Color scheme consistent with Figure 5(a)

colors = [

0.2 0.6 0.8; % Blue - Standard

0.2 0.8 0.4; % Green - Low temp

0.9 0.3 0.3; % Red - High temp

0.5 0.5 0.5	% Gray - Envelope

];

%% Create figure with 2×2 small multiples layout

figure('Name', 'UPT Efficiency Characterization - Small Multiples', ...

'Position', [500 200 1000 700], 'Color', 'w');

tiledlayout(2, 2, 'TileSpacing', 'compact', 'Padding', 'compact');

% Distance range for UPT hover scenario (5-35m)

d_up = linspace(5, 35, 200);

% Store standard condition curve for envelope comparison

alpha_std_lin = (alpha0 / 4.343);

eta_air_std = exp(-alpha_std_lin .* d_up);

eta_UPT_std = eta_trans .* eta_air_std .* eta_rect * 100;

%% Subplot 1: Standard Condition

nexttile;

alpha = alpha0;

alpha_lin = alpha / 4.343;

eta_air = exp(-alpha_lin .* d_up);

eta_UPT = eta_trans .* eta_air .* eta_rect * 100;

plot(d_up, eta_UPT, '-', 'LineWidth', 2.5, 'Color', colors(1,:));

hold on;

% Mark key operating points from Table 4

d_mark = [10, 20, 30];

eta_mark = eta_trans * exp(-alpha_std_lin * d_mark) * eta_rect * 100;

plot(d_mark, eta_mark, 'o', 'MarkerSize', 10, 'MarkerFaceColor', colors(1,:), ...

'MarkerEdgeColor', 'k', 'LineWidth', 1.5);

% Annotate values

for k = 1:length(d_mark)

text(d_mark(k), eta_mark(k) + 2, sprintf('%.1f%%', eta_mark(k)), ...

'FontSize', 9, 'FontWeight', 'bold', 'HorizontalAlignment', 'center');

end

% Min hover power threshold (50W -> 10% efficiency)

eta_threshold = 50 / Ptx_US * 100;

plot([5 35], [eta_threshold eta_threshold], 'g-.', 'LineWidth', 1.5);

text(28, eta_threshold + 1.5, 'Min Hover Power (50W)', ...

'Color', [0 0.5 0], 'FontSize', 9);

xlabel('Distance d_{i,j} (m)', 'FontSize', 10, 'FontWeight', 'bold');

ylabel('UPT Efficiency \eta_{UPT} (%)', 'FontSize', 10, 'FontWeight', 'bold');

title('(a) Standard Condition (20°C, 50%RH)', 'FontSize', 11, 'FontWeight', 'bold');

xlim([5 35]); ylim([0 45]); grid on;

% Parameter annotation

alpha = 0.9;

eta_UPT = [38.6];

str = ['\alpha = ' num2str(alpha, '%.2f') ' dB/m' newline ...

'\eta_{UPT}^{max} = ' num2str(eta_UPT(1), '%.1f') '%'];

text(0.05, 0.95, str, 'Units', 'normalized', 'VerticalAlignment', 'top', ...

'FontSize', 9, 'BackgroundColor', [1 1 0.9], 'EdgeColor', 'k', ...

'Interpreter', 'tex');%% Subplot 2: Low Temperature (Best Case)

nexttile;

T = 5; RH = 30;

alpha = alpha0 * (1 + 0.008*(T - T_std)) * (1 + 0.005*(RH - RH_std));

alpha_lin = alpha / 4.343;

eta_air = exp(-alpha_lin .* d_up);

eta_UPT = eta_trans .* eta_air .* eta_rect * 100;

plot(d_up, eta_UPT, '-', 'LineWidth', 2.5, 'Color', colors(2,:));

hold on;

% Dim standard condition as reference

plot(d_up, eta_UPT_std, '--', 'LineWidth', 1, 'Color', colors(1,:), ...

'HandleVisibility', 'off');

% Mark operating points

eta_mark_low = eta_trans * exp(-alpha_lin * d_mark) * eta_rect * 100;

plot(d_mark, eta_mark_low, 's', 'MarkerSize', 10, 'MarkerFaceColor', colors(2,:), ...

'MarkerEdgeColor', 'k', 'LineWidth', 1.5);

for k = 1:length(d_mark)

text(d_mark(k), eta_mark_low(k) + 2, sprintf('%.1f%%', eta_mark_low(k)), ...

'FontSize', 9, 'FontWeight', 'bold', 'HorizontalAlignment', 'center');

end

plot([5 35], [eta_threshold eta_threshold], 'g-.', 'LineWidth', 1.5);

xlabel('Distance d_{i,j} (m)', 'FontSize', 10, 'FontWeight', 'bold');

ylabel('UPT Efficiency \eta_{UPT} (%)', 'FontSize', 10, 'FontWeight', 'bold');

title('(b) Low Temp/Low Humidity (5°C, 30%RH)', 'FontSize', 11, 'FontWeight', 'bold');

xlim([5 35]); ylim([0 45]); grid on;

str = ['\alpha = ' num2str(alpha, '%.2f') ' dB/m' newline ...

'\eta_{UPT}^{max} = ' num2str(eta_UPT(1), '%.1f') '%'];

text(0.05, 0.95, str, 'Units', 'normalized', 'VerticalAlignment', 'top', ...

'FontSize', 9, 'BackgroundColor', [1 1 0.9], 'EdgeColor', 'k', ...

'Interpreter', 'tex');

%% Subplot 3: High Temperature (Worst Case)

nexttile;

T = 35; RH = 80;

alpha = alpha0 * (1 + 0.008*(T - T_std)) * (1 + 0.005*(RH - RH_std));

alpha_lin = alpha / 4.343;

eta_air = exp(-alpha_lin .* d_up);

eta_UPT = eta_trans .* eta_air .* eta_rect * 100;

plot(d_up, eta_UPT, '-', 'LineWidth', 2.5, 'Color', colors(3,:));

hold on;

% Dim standard condition as reference

plot(d_up, eta_UPT_std, '--', 'LineWidth', 1, 'Color', colors(1,:), ...

'HandleVisibility', 'off');

% Mark operating points

eta_mark_high = eta_trans * exp(-alpha_lin * d_mark) * eta_rect * 100;

plot(d_mark, eta_mark_high, '^', 'MarkerSize', 10, 'MarkerFaceColor', colors(3,:), ...

'MarkerEdgeColor', 'k', 'LineWidth', 1.5);

for k = 1:length(d_mark)

text(d_mark(k), eta_mark_high(k) + 2, sprintf('%.1f%%', eta_mark_high(k)), ...

'FontSize', 9, 'FontWeight', 'bold', 'HorizontalAlignment', 'center');

end

plot([5 35], [eta_threshold eta_threshold], 'g-.', 'LineWidth', 1.5);

xlabel('Distance d_{i,j} (m)', 'FontSize', 10, 'FontWeight', 'bold');

ylabel('UPT Efficiency \eta_{UPT} (%)', 'FontSize', 10, 'FontWeight', 'bold');

title('(c) High Temp/High Humidity (35°C, 80%RH)', 'FontSize', 11, 'FontWeight', 'bold');

xlim([5 35]); ylim([0 45]); grid on;

text(0.05, 0.95, ['\alpha = ' num2str(alpha, '%.2f') ' dB/m' newline ...

'\eta_{UPT}^{max} = ' num2str(eta_UPT(1), '%.1f') '%'], ...

'Units', 'normalized', ...

'VerticalAlignment', 'top', ...

'FontSize', 9, ...

'BackgroundColor', [1 1 0.9], ...

'EdgeColor', 'k', ...

'Interpreter', 'tex');

%% Subplot 4: Full Operational Envelope

nexttile;

% Calculate envelope boundaries

alpha_min = alpha0 * (1 + 0.008*(5 - 20)) * (1 + 0.005*(30 - 50)); % Best case

alpha_max = alpha0 * (1 + 0.008*(35 - 20)) * (1 + 0.005*(80 - 50)); % Worst case

alpha_min_lin = alpha_min / 4.343;

alpha_max_lin = alpha_max / 4.343;

eta_air_best = exp(-alpha_min_lin .* d_up);

eta_air_worst = exp(-alpha_max_lin .* d_up);

eta_UPT_best = eta_trans .* eta_air_best .* eta_rect * 100;

eta_UPT_worst = eta_trans .* eta_air_worst .* eta_rect * 100;

% Fill operational envelope

fill([d_up, fliplr(d_up)], [eta_UPT_best, fliplr(eta_UPT_worst)], ...

[0.85 0.92 0.98], 'EdgeColor', 'none', 'FaceAlpha', 0.5);

hold on;

% Plot boundary curves

plot(d_up, eta_UPT_best, '-', 'LineWidth', 2, 'Color', colors(2,:));

plot(d_up, eta_UPT_worst, '-', 'LineWidth', 2, 'Color', colors(3,:));

plot(d_up, eta_UPT_std, '-', 'LineWidth', 2.5, 'Color', colors(1,:));

% Mark operating points for all conditions

plot(d_mark, eta_mark, 'o', 'MarkerSize', 8, 'MarkerFaceColor', colors(1,:), ...

'MarkerEdgeColor', 'k', 'LineWidth', 1.5);

plot(d_mark, eta_mark_low, 's', 'MarkerSize', 8, 'MarkerFaceColor', colors(2,:), ...

'MarkerEdgeColor', 'k', 'LineWidth', 1.5);

plot(d_mark, eta_mark_high, '^', 'MarkerSize', 8, 'MarkerFaceColor', colors(3,:), ...

'MarkerEdgeColor', 'k', 'LineWidth', 1.5);

% Threshold line

plot([5 35], [eta_threshold eta_threshold], 'g-.', 'LineWidth', 1.5);

% Legend

legend({'Operational Envelope', 'Best Case (5°C, 30%RH)', ...

'Worst Case (35°C, 80%RH)', 'Standard (20°C, 50%RH)', ...

'Standard Points', 'Best Points', 'Worst Points', 'Min Power Threshold'}, ...

'Location', 'northeast', 'FontSize', 8);

xlabel('Distance d_{i,j} (m)', 'FontSize', 10, 'FontWeight', 'bold');

ylabel('UPT Efficiency \eta_{UPT} (%)', 'FontSize', 10, 'FontWeight', 'bold');

title('(d) Operational Envelope (All Conditions)', 'FontSize', 11, 'FontWeight', 'bold');

xlim([5 35]); ylim([0 45]); grid on;

% Envelope statistics

text(0.05, 0.95, sprintf('Envelope Range:\n%.1f%% - %.1f%% @ 10m\n%.1f%% - %.1f%% @ 30m', ...

eta_UPT_best(1), eta_UPT_worst(1), ...

eta_UPT_best(end), eta_UPT_worst(end)), ...

'Units', 'normalized', ... % 这设置的是 text 框的单位，不是位置！

'VerticalAlignment', 'top', ...

'FontSize', 9, ...

'BackgroundColor', [1 1 0.9], ...

'EdgeColor', 'k');

%% Overall title

% sgtitle('Figure 6: UPT Efficiency Characterization and Outdoor Robustness', ...

%	'FontSize', 14, 'FontWeight', 'bold');

%% Save figure

print('-dpng', '-r300', 'Figure 6_UPT_Efficiency.png');

fprintf('Figure 6 generated successfully.\n');

% Print verification data

fprintf('\nVerification against Section 2.3.3 Table 4:\n');

fprintf('Standard Condition (20°C, 50%%RH, alpha=%.2f dB/m):\n', alpha0);

fprintf(' @ 10m: eta_UPT = %.1f%% (expected: 38.6%%)\n', eta_mark(1));

fprintf(' @ 30m: eta_UPT = %.1f%% (expected: 12.4%%)\n', eta_mark(3));

fprintf(' Received Power @ 10m: %.0f W (expected: 193W)\n', Ptx_US * eta_mark(1)/100);

fprintf(' Received Power @ 30m: %.0f W (expected: 62W)\n', Ptx_US * eta_mark(3)/100);

% =========================================================================

% Figure 3

% =========================================================================

t = 0:0.25:24; % 15-minute sampling, 96 points total

lambda_peak = 0.72; % Carbon emission peak kg/kWh

lambda_valley = 0.42; % Carbon emission valley kg/kWh

T = 24;

% Piecewise function construction

lambda_t = zeros(size(t));

day_period = (t >= 6) & (t <= 22);

night_period = ~day_period;

lambda_t(day_period) = lambda_valley + 0.5*(lambda_peak - lambda_valley) * ...

(1 - cos(2*pi*(t(day_period)-6)/16));

lambda_t(night_period) = lambda_valley;

% Add white noise

rng(42);

lambda_t = lambda_t + 0.02 * randn(size(t));

% Plotting

figure('Name','Dynamic Carbon Emission Factor Model','Position',[100 100 800 500]);

plot(t, lambda_t, 'b-', 'LineWidth', 2);

hold on;

fill([6 22 22 6], [lambda_valley lambda_valley lambda_peak lambda_peak], ...

'b', 'FaceAlpha', 0.1, 'EdgeColor', 'none');

plot([6 6], [0.4 0.75], 'r--', 'LineWidth', 1.5);

plot([22 22], [0.4 0.75], 'r--', 'LineWidth', 1.5);

xlabel('Time (h)', 'FontSize', 12);

ylabel('Carbon Emission Factor λ(t) (kg/kWh)', 'FontSize', 12);

title('24-Hour Dynamic Carbon Emission Factor Curve (Peak-Valley Difference 42%)', 'FontSize', 14);

grid on; box on;

legend('Carbon Emission Factor λ(t)', 'Daytime Operation Zone', 'Confidence Interval Boundary');

text(8, 0.7, 'Peak Period: 0.72 kg/kWh', 'FontSize', 10);

text(18, 0.45, 'Valley Period: 0.42 kg/kWh', 'FontSize', 10);

% =========================================================================

% Figure 4

% =========================================================================

flight_states = {'Taxiing', 'Vertical Takeoff', 'Climb', 'Cruise', 'Approach & Landing'};

power_kW = [18, 220, 180, 120, 160]; % Average power (kW)

duration_s = [20, 30, 90, 540, 60]; % Duration (seconds)

energy_kWh = power_kW .* duration_s / 3600; % Energy calculation (kWh)

% Create segmented area chart

figure('Name','eVTOL Energy Consumption Profile','Position',[200 200 900 500]);

cmap = autumn(5);

hold on;

y_cumsum = zeros(size(t));

t_cumulative = cumsum([0, duration_s(1:end-1)]);

for i = 1:5

area_i = energy_kWh(i) * ones(1, duration_s(i));

t_segment = t_cumulative(i) + (0:duration_s(i)-1);

fill([t_segment, fliplr(t_segment)], ...

[zeros(size(area_i)), fliplr(area_i)], ...

cmap(i,:), 'FaceAlpha', 0.7, 'EdgeColor', 'none');

text(t_cumulative(i) + duration_s(i)/2, energy_kWh(i)/2, ...

sprintf('%s\n%.1f kWh', flight_states{i}, energy_kWh(i)), ...

'HorizontalAlignment', 'center', 'FontSize', 9);

end

xlabel('Flight Time (s)', 'FontSize', 12);

ylabel('Energy Consumption (kWh)', 'FontSize', 12);

title('eVTOL Single Mission Energy Profile Decomposition (Total: 27.1 kWh)', 'FontSize', 14);

xlim([0 740]); ylim([0 30]);

grid on; box on;

% =========================================================================

% Figure 5

% =========================================================================

P_static = 1.2; % kW (Static power consumption)

P_active = 2.8; % kW (Including sensing and wireless energy transfer)

E_sw = 25; % J (Switching energy cost for wake-up)

N_bts = 64; % Total number of base stations

T_cycle = 3600; % 1-hour scheduling cycle

Awake_time = linspace(0, T_cycle, 100); % Awake duration within a cycle

% Calculate energy consumption under different sleep ratios

sleep_ratio = 1 - Awake_time/T_cycle;

Energy_per_cycle = (P_static + sleep_ratio * (P_active - P_static)) * T_cycle/1000 ...

+ E_sw * N_bts * (sleep_ratio > 0 & sleep_ratio < 1);

% Normalization

Energy_normalized = Energy_per_cycle / max(Energy_per_cycle);

% Plotting

figure('Name','BS Sleep Energy Efficiency','Position',[300 200 800 500]);

yyaxis left;

plot(sleep_ratio*100, Energy_normalized*100, 'b-o', 'LineWidth', 2);

ylabel('Relative Energy Consumption (%)', 'FontSize', 12);

ylim([60 100]);

yyaxis right;

plot(sleep_ratio*100, sleep_ratio*100, 'r--', 'LineWidth', 2);

ylabel('BS Sleep Ratio (%)', 'FontSize', 12);

ylim([0 100]);

xlabel('Sleep Ratio (%)', 'FontSize', 12);

title('Trade-off Between BS Sleep Ratio and System Energy Consumption', 'FontSize', 14);

grid on; box on;

legend('Total System Energy', 'Linear Reference of Sleep Ratio', 'Location', 'best');

% Annotate key point

hold on;

plot(38, 73, 'ko', 'MarkerSize', 10, 'LineWidth', 2);

text(38, 73, 'Optimal Sleep Point\n38% Sleep → 27% Energy Saving', ...

'HorizontalAlignment', 'center', 'FontSize', 10, 'Color', 'k');

% =========================================================================

% Figure 6-7

% =========================================================================

clc; close all; clear;

%% Parameter Settings (from Section 2.3.3)

EIRP_dBm = 50; % Equivalent Isotropic Radiated Power (dBm)

Pt = 10^(EIRP_dBm/10) * 1e-3; % Transmit power (W)

Gt = 10^(18/10); % Transmit antenna gain (18 dBi)

Gr = 10^(12/10); % Receive antenna gain (12 dBi)

lambda = 0.0857; % Wavelength at 3.5 GHz (m)

eta_max = 0.85; % Maximum rectification efficiency

P_th = 0.1; % Threshold power (W)

k = 2.5; % Steepness coefficient (W^-1)

% Define grid ranges

d = linspace(1, 50, 200); % Distance (m), 1 to 50

theta_deg = linspace(0, 60, 200); % Deviation angle (degrees), 0 to 60

theta = deg2rad(theta_deg); % Convert to radians

[D, THETA] = meshgrid(d, theta);

THETA_DEG = rad2deg(THETA);

%% Calculate Received Power using Friis Equation with Angle Dependence

% Free-space path loss

FSPL = (lambda ./ (4*pi*D)).^2;

% Angle-dependent gain reduction (Gaussian beam pattern)

theta_3dB = deg2rad(15); % 3-dB beamwidth (15 degrees)

G_pattern = exp(-2.773 * (THETA/theta_3dB).^2);

% Effective antenna gain

G_eff = Gt * Gr * G_pattern;

% Received power (W) - Equation (12) in paper

Pr = Pt * G_eff .* FSPL;

% Practical degradation factors (Section 2.3.3)

L_cable = 10^(-3/10); % 3 dB cable/feed loss

L_mismatch = 10^(-2/10); % 2 dB impedance mismatch

L_aperture = 0.40; % 40% effective aperture utilization

L_attitude = 10^(-2/10); % 2 dB attitude-induced pointing loss

L_regulatory = 10^(-3/10); % 3 dB below theoretical EIRP

% Composite practical factor

L_practical = L_cable * L_mismatch * L_aperture * L_attitude * L_regulatory;

Pr_practical = Pr * L_practical;

%% Calculate Rectification Efficiency (Logistic Model) - Equation (14)

eta_rect = eta_max ./ (1 + exp(-k*(Pr - P_th)));

eta_rect_practical = eta_max ./ (1 + exp(-k*(Pr_practical - P_th)));

%% Create Enhanced 3D Surface Plot with Contour Projection

figure('Position', [100 100 1000 750], 'Color', 'w', 'Renderer', 'zbuffer');

hold on;

% ========== MAIN SURFACE PLOT ==========

% Plot surface with fine colormap

surf(D, THETA_DEG, eta_rect*100, ...

'EdgeColor', 'none', ...

'FaceAlpha', 0.85, ...

'FaceColor', 'interp');

% ========== CONTOUR LINES ON BOTTOM PROJECTION PLANE ==========

% Define contour levels for efficiency (%)

contour_levels = [30, 50, 70, 85];

% Project contour lines onto bottom plane (z = 0)

% Note: For R2018a compatibility, use separate contour call without clabel

% First, draw the contour lines on the surface

[C, h_contour] = contour3(D, THETA_DEG, eta_rect*100, ...

contour_levels, ...

'LineWidth', 2.0, ...

'Color', [0.15 0.15 0.15], ...

'LineStyle', '--');

% Highlight critical 70% efficiency boundary (high-efficiency zone threshold)

contour3(D, THETA_DEG, eta_rect*100, [70 70], ...

'LineWidth', 3.0, ...

'Color', [0.85 0.15 0.15], ...

'LineStyle', '-');

% Highlight 30% efficiency boundary (ultrasonic switching threshold)

contour3(D, THETA_DEG, eta_rect*100, [30 30], ...

'LineWidth', 2.5, ...

'Color', [0.9 0.5 0.1], ...

'LineStyle', '-.');

% ========== BOTTOM PROJECTION PLANE ==========

% Create semi-transparent plane at z = 0 for contour visibility

% For R2018a, use fill3 instead of patch with FaceAlpha

h_plane = fill3([1 50 50 1], [0 0 60 60], [0 0 0 0], [0.92 0.92 0.92]);

set(h_plane, 'FaceAlpha', 0.25, 'EdgeColor', [0.7 0.7 0.7], 'LineWidth', 0.5);

% ========== CONTOUR LABELS (R2018a Compatible) ==========

% Use text annotations instead of clabel for R2018a compatibility

% Manually place labels at representative points along contours

text(10, 5, 0, '85%', 'FontSize', 10, 'FontWeight', 'bold', ...

'Color', [0.1 0.1 0.1], 'HorizontalAlignment', 'center', ...

'BackgroundColor', [1 1 1]); % RGB only, no alpha

text(15, 12, 0, '70%', 'FontSize', 10, 'FontWeight', 'bold', ...

'Color', [0.85 0.15 0.15], 'HorizontalAlignment', 'center', ...

'BackgroundColor', [1 1 1]);

text(22, 20, 0, '50%', 'FontSize', 10, 'FontWeight', 'bold', ...

'Color', [0.1 0.1 0.1], 'HorizontalAlignment', 'center', ...

'BackgroundColor', [1 1 1]);

text(30, 30, 0, '30%', 'FontSize', 10, 'FontWeight', 'bold', ...

'Color', [0.9 0.5 0.1], 'HorizontalAlignment', 'center', ...

'BackgroundColor', [1 1 1]);

% ========== COLORBAR WITH INCREASED FONT SIZE ==========

colormap(jet(256));

c = colorbar('Location', 'eastoutside');

c.Label.String = 'Rectification Efficiency \eta_{rect} (%)';

c.Label.FontSize = 12; % Increased from default 9 pt

c.Label.FontWeight = 'bold';

c.Ticks = [0 10 20 30 40 50 60 70 80 90 100];

c.TickLabels = {'0', '10', '20', '30', '40', '50', '60', '70', '80', '90', '100'};

c.FontSize = 11; % Increased from default 9 pt

% ========== AXIS LABELS WITH INCREASED FONT SIZE ==========

xlabel('Distance d (m)', ...

'FontSize', 12, ... % Increased from 9 pt to 12 pt

'FontWeight', 'bold', ...

'Color', [0.1 0.1 0.1]);

ylabel('Deviation Angle \theta (°)', ...

'FontSize', 12, ... % Increased from 9 pt to 12 pt

'FontWeight', 'bold', ...

'Color', [0.1 0.1 0.1]);

zlabel('Rectification Efficiency \eta_{rect} (%)', ...

'FontSize', 12, ... % Increased from 9 pt to 12 pt

'FontWeight', 'bold', ...

'Color', [0.1 0.1 0.1]);

% ========== TITLE WITH INCREASED FONT SIZE ==========

% title({'Wireless Charging Efficiency vs Distance and Angle'; ...

%	'(EIRP = 50 dBm, A_r = 0.02 m^2, Ideal Conditions)'}, ...

%	'FontSize', 13, ... % Increased from 10 pt to 13 pt

%	'FontWeight', 'bold', ...

%	'Color', [0.1 0.1 0.1]);

% ========== ANNOTATIONS FOR HIGH-EFFICIENCY ZONE ==========

% Text annotation for high-efficiency zone

text(8, 8, 95, 'High-Efficiency Zone', ...

'FontSize', 11, ...

'FontWeight', 'bold', ...

'Color', [0.2 0.6 0.2]);

text(8, 12, 88, '\eta_{rect} > 70%', ...

'FontSize', 10, ...

'Color', [0.2 0.6 0.2]);

% Boundary markers

text(15, 15, 72, '70% Boundary', ...

'FontSize', 9, ...

'Color', [0.85 0.15 0.15], ...

'FontWeight', 'bold');

% ========== ANNOTATIONS FOR SWITCHING THRESHOLD ==========

text(35, 45, 35, 'Ultrasonic Switching', ...

'FontSize', 10, ...

'FontWeight', 'bold', ...

'Color', [0.9 0.5 0.1]);

text(35, 48, 28, 'Threshold: \eta < 30%', ...

'FontSize', 9, ...

'Color', [0.9 0.5 0.1]);

% ========== VIEW AND LIGHTING SETTINGS ==========

view(45, 30); % Azimuth 45°, Elevation 30°

grid on;

box on;

% Enhanced lighting for better 3D perception

light('Position', [50 60 100], 'Style', 'infinite');

lighting gouraud;

material dull;

% ========== AXIS LIMITS AND TICKS ==========

xlim([1 50]);

ylim([0 60]);

zlim([0 100]);

xticks([0 10 20 30 40 50]);

yticks([0 15 30 45 60]);

zticks([0 20 40 60 80 100]);

% ========== AXIS TICK FONT SIZES ==========

set(gca, 'FontSize', 11, ... % Increased from default 9 pt

'LineWidth', 1.2, ...

'XColor', [0.1 0.1 0.1], ...

'YColor', [0.1 0.1 0.1], ...

'ZColor', [0.1 0.1 0.1], ...

'GridLineStyle', '--', ...

'GridAlpha', 0.3);

% ========== LEGEND ==========

legend('Surface', 'Contour Lines (30%, 50%, 70%, 85%)', ...

'70% High-Efficiency Boundary', '30% Switching Threshold', ...

'Bottom Projection Plane', ...

'Location', 'northwest', ...

'FontSize', 10, ...

'Color', [1 1 1]);

hold off;

%% ========== EXPORT HIGH-RESOLUTION FIGURE ==========

print('-dpng', '-r600', 'Figure6_Enhanced_Wireless_Charging_Efficiency.png');

print('-depsc', '-r600', 'Figure6_Enhanced_Wireless_Charging_Efficiency.eps');

disp('Enhanced Figure 6 generated successfully with:');

disp(' - Contour lines on bottom projection plane (z=0)');

disp(' - Contour levels: 30%, 50%, 70%, 85%');

disp(' - 70% boundary highlighted in red (3 pt line width)');

disp(' - 30% switching threshold in orange dash-dot');

disp(' - Axis labels: 12 pt (bold)');

disp(' - Colorbar labels: 11 pt');

disp(' - Contour labels: 10 pt (bold) via text annotations');

disp(' - Axis ticks: 11 pt');

disp(' - R2018a compatible (no RGBA in BackgroundColor)');

%% ========== OPTIONAL: PRACTICAL CONDITIONS SUBPLOT ==========

figure('Position', [150 150 1200 500], 'Color', 'w');

% Subplot 1: Ideal conditions

subplot(1, 2, 1);

surf(D, THETA_DEG, eta_rect*100, 'EdgeColor', 'none', 'FaceAlpha', 0.85);

hold on;

contour3(D, THETA_DEG, eta_rect*100, [30 50 70 85], ...

'LineWidth', 1.5, 'Color', [0.2 0.2 0.2], 'LineStyle', '--');

contour3(D, THETA_DEG, eta_rect*100, [70 70], ...

'LineWidth', 2.5, 'Color', [0.85 0.15 0.15]);

xlabel('Distance d (m)', 'FontSize', 11, 'FontWeight', 'bold');

ylabel('Deviation Angle \theta (°)', 'FontSize', 11, 'FontWeight', 'bold');

zlabel('Efficiency (%)', 'FontSize', 11, 'FontWeight', 'bold');

% title('(a) Ideal Conditions (50 dBm EIRP)', 'FontSize', 12, 'FontWeight', 'bold');

set(gca, 'FontSize', 10);

colorbar('FontSize', 10);

view(45, 30);

grid on;

% Subplot 2: Practical conditions

subplot(1, 2, 2);

surf(D, THETA_DEG, eta_rect_practical*100, 'EdgeColor', 'none', 'FaceAlpha', 0.85);

hold on;

contour3(D, THETA_DEG, eta_rect_practical*100, [10 20 30 50], ...

'LineWidth', 1.5, 'Color', [0.2 0.2 0.2], 'LineStyle', '--');

contour3(D, THETA_DEG, eta_rect_practical*100, [30 30], ...

'LineWidth', 2.5, 'Color', [0.85 0.15 0.15]);

xlabel('Distance d (m)', 'FontSize', 11, 'FontWeight', 'bold');

ylabel('Deviation Angle \theta (°)', 'FontSize', 11, 'FontWeight', 'bold');

zlabel('Efficiency (%)', 'FontSize', 11, 'FontWeight', 'bold');

% title('(b) Practical Conditions (47 dBm EIRP, 5 dB Loss)', 'FontSize', 12, 'FontWeight', 'bold');

set(gca, 'FontSize', 10);

colorbar('FontSize', 10);

view(45, 30);

grid on;

% For R2018a, use annotation instead of sgtitle

% annotation('textbox', [0.3 0.95 0.4 0.05], 'String', ...

%	'Wireless Charging Efficiency: Ideal vs Practical', ...

%	'FontSize', 14, 'FontWeight', 'bold', 'EdgeColor', 'none', ...

%	'HorizontalAlignment', 'center');

print('-dpng', '-r600', 'Figure6_Comparison_Ideal_vs_Practical.png');

disp('Comparison figure (Ideal vs Practical) generated successfully.');

% =========================================================================

% Figure 8

% =========================================================================

schemes = {'Fixed Charging Pile', 'GNN Sleep Only', 'PSO Only', 'GNN-PSO'};

figure('Name', 'Scheme Comparison - Small Multiples', 'Position', [500 200 1000 700]);

% 2×2 matrix layout with shared axes

tiledlayout(2, 2, 'TileSpacing', 'compact');

for i = 1:4

nexttile;

% Draw the primary scheme

values = [HV_norm(i), delay_norm(i), time_norm(i), HV_norm(i)];

polarplot(angles, values, 'o-', 'LineWidth', 2.5, 'MarkerSize', 8, ...

'Color', colors(i,:));

% Dim other schemes as background reference

hold on;

for j = setdiff(1:4, i)

other_val = [HV_norm(j), delay_norm(j), time_norm(j), HV_norm(j)];

polarplot(angles, other_val, '--', 'LineWidth', 1, ...

'Color', colors(j,:), 'HandleVisibility', 'off');

end

rlim([0 100]);

title(schemes{i}, 'FontSize', 12, 'FontWeight', 'bold');

thetaticklabels({});

% Add scheme-specific metric annotation

text(0, 0, sprintf('Pareto HV\n%.2f', HV(i)), 'HorizontalAlignment', 'center', ...

'FontSize', 10, 'Color', colors(i,:));

end

% Overall title and legend

%sgtitle('Four-Scheme Radar Chart Comparison Matrix', 'FontSize', 16, 'FontWeight', 'bold');

lgd = legend(schemes, 'Location', 'southoutside', 'Orientation', 'horizontal');

lgd.Layout.Tile = 'south'; % Place legend at the bottom

% =========================================================================

% Figure 9

% =========================================================================

sleep_rates = [0, 10, 20, 30, 38, 45, 50];

energy_saving = [0, 8.2, 15.5, 22.1, 27.4, 29.8, 31.2]; % % Energy Saving

carbon_reduction = [0, 8.8, 16.8, 24.2, 28.9, 31.5, 33.1]; % % Carbon Reduction

% Annual Emission Reduction Roadmap

years = 2025:2030;

sleep_target = [20, 25, 30, 35, 38, 45];

annual_flights = linspace(1e5, 5e5, length(years)); % 100k to 500k flights

CO2_reduction = 0.92 * sleep_target/38 .* annual_flights; % kg

trees_equivalent = CO2_reduction / 5.9; % Annual carbon sequestration per tree: 5.9 kg

% Dual-axis Figure

figure('Name','Pareto Frontier and Emission Reduction Roadmap','Position',[600 200 1200 500]);

% Subplot 1: Pareto Frontier

subplot(1,2,1);

plot(energy_saving, carbon_reduction, 'b-o', 'LineWidth', 2, 'MarkerSize', 8);

hold on;

plot(27.4, 28.9, 'ro', 'MarkerSize', 12, 'LineWidth', 3);

text(27.4, 28.9, ' Optimal Operating Point\n (38% Sleep)', 'VerticalAlignment', 'bottom');

xlabel('Energy Saving per Flight (%)', 'FontSize', 12);

ylabel('Carbon Reduction Effect (%)', 'FontSize', 12);

title('Energy-Carbon Pareto Frontier', 'FontSize', 14);

grid on; box on;

legend('Pareto Frontier', 'Actual Optimal Solution');

annotation('textarrow', [0.6 0.7], [0.35 0.45], 'String', 'Non-dominated Solution Set');

% Subplot 2: Emission Reduction Roadmap

subplot(1,2,2);

yyaxis left;

bar(years, CO2_reduction/1e3, 'b', 'FaceAlpha', 0.7);

ylabel('Annual CO? Reduction (kt)', 'FontSize', 12);

ylim([0 25]);

yyaxis right;

plot(years, trees_equivalent/1e4, 'r-s', 'LineWidth', 2, 'MarkerSize', 6);

ylabel('Equivalent Trees Planted (×10k)', 'FontSize', 12);

ylim([0 30]);

xlabel('Year', 'FontSize', 12);

title('2025-2030 Low-Altitude Economy Emission Reduction Roadmap', 'FontSize', 14);

grid on;

legend('CO? Reduction', 'Equivalent Tree Planting', 'Location', 'northwest');
